# Imunofan—RDKVYR Peptide—Stimulates Skin Cell Proliferation and Promotes Tissue Repair

**DOI:** 10.3390/molecules25122884

**Published:** 2020-06-23

**Authors:** Justyna Sawicka, Maria Dzierżyńska, Anna Wardowska, Milena Deptuła, Piotr Rogujski, Paweł Sosnowski, Natalia Filipowicz, Alina Mieczkowska, Piotr Sass, Anna Pawlik, Aleksandra Hać, Adriana Schumacher, Magdalena Gucwa, Natalia Karska, Jolanta Kamińska, Rafał Płatek, Jarosław Mazuryk, Jacek Zieliński, Karolina Kondej, Piotr Młynarz, Piotr Mucha, Piotr Skowron, Łukasz Janus, Anna Herman-Antosiewicz, Paweł Sachadyn, Artur Czupryn, Arkadiusz Piotrowski, Michał Pikuła, Sylwia Rodziewicz-Motowidło

**Affiliations:** 1 Department of Biomedical Chemistry, Faculty of Chemistry, University of Gdańsk, 80-308 Gdańsk, Poland; justyna.sawicka@ug.edu.pl (J.S.); maria.dzierzynska@ug.edu.pl (M.D.); natalia.karska@ug.edu.pl (N.K.); 2 Laboratory of Tissue Engineering and Regenerative Medicine, Department of Embryology, Medical University of Gdańsk, 80-210 Gdańsk, Poland; anna.wardowska@gumed.edu.pl (A.W.); milenadeptula@gumed.edu.pl (M.D.); 3 Laboratory of Neurobiology, Nencki Institute of Experimental Biology, Polish Academy of Sciences, 02-093 Warsaw, Poland; progujski@imdik.pan.pl (P.R.); rplatek1982@gmail.com (R.P.); jmazuryk@ichf.edu.pl (J.M.); artur@nencki.gov.pl (A.C.); 4 NeuroRepair Department, Mossakowski Medical Research Centre, Polish Academy of Sciences, 02-106 Warsaw, Poland; 5 Laboratory for Regenerative Biotechnology, Faculty of Chemistry, Gdańsk University of Technology, 80-233 Gdańsk, Poland; paw.sosno@gmail.com (P.S.); piotrsass@gmail.com (P.S.); jolantakaminska6@gmail.com (J.K.); psach@pg.edu.pl (P.S.); 6 Department of Biology and Pharmaceutical Botany, Faculty of Pharmacy, Medical University of Gdańsk, 80-416 Gdańsk, Poland; nata@gumed.edu.pl (N.F.); alina_mieczkowska@gumed.edu.pl (A.M.); magdag@gumed.edu.pl (M.G.); arpiotr@gumed.edu.pl (A.P.); 7 International Research Agenda 3P-Medicine Laboratory, Medical University of Gdańsk, 80-210 Gdańsk, Poland; 8 Department of Medical Biology and Genetics, Faculty of Biology, University of Gdańsk, 80-308 Gdańsk, Poland; anna.pawlik@biol.ug.edu.pl (A.P.); aleksandra.wiczk@biol.ug.edu.pl (A.H.); anna.herman@biol.ug.edu.pl (A.H.-A.); 9 Department of Embryology, Medical University of Gdańsk, 80-211 Gdańsk, Poland; aschumacher@gumed.edu.pl; 10 Institute of Physical Chemistry, Polish Academy of Sciences, 01-224 Warsaw, Poland; 11 Department of Surgical Oncology, Medical University of Gdańsk, 80-210 Gdańsk, Poland; jacek.zielinski@gumed.edu.pl; 12 Department of Plastic Surgery, Medical University of Gdańsk, 80-210 Gdańsk, Poland; kondej@gumed.edu.pl; 13 Department of Bioorganic Chemistry, Wrocław University of Technology, 50-370 Wrocław, Poland; piotr.mlynarz@pwr.edu.pl; 14 Department of Molecular Biochemistry, Faculty of Chemistry, University of Gdańsk80-308 Gdańsk, Poland; piotr.mucha@ug.edu.pl; 15 Department of Molecular Biotechnology, Faculty of Chemistry, University of Gdańsk, 80-308 Gdańsk, Poland; piotr.skowron@ug.edu.pl; 16 MedVentures Company, 60-141 Poznań, Poland; j.medventures@gmail.com

**Keywords:** imunofan, peptides, cell proliferation, fibroblasts, keratinocytes, adipose-derived stem cells, transcriptomics, immunological safety, wound healing, ear pinna model

## Abstract

Regeneration and wound healing are vital to tissue homeostasis and organism survival. One of the biggest challenges of today’s science and medicine is finding methods and factors to stimulate these processes in the human body. Effective solutions to promote regenerative responses will accelerate advances in tissue engineering, regenerative medicine, transplantology, and a number of other clinical specialties. In this study, we assessed the potential efficacy of a synthetic hexapeptide, RDKVYR, for the stimulation of tissue repair and wound healing. The hexapeptide is marketed under the name “Imunofan” (IM) as an immunostimulant. IM displayed stability in aqueous solutions, while in plasma it was rapidly bound by albumins. Structural analyses demonstrated the conformational flexibility of the peptide. Tests in human fibroblast and keratinocyte cell lines showed that IM exerted a statistically significant (*p* < 0.05) pro-proliferative activity (30–40% and 20–50% increase in proliferation of fibroblast and keratinocytes, respectively), revealed no cytotoxicity over a vast range of concentrations (*p* < 0.05), and had no allergic properties. IM was found to induce significant transcriptional responses, such as enhanced activity of genes involved in active DNA demethylation (*p* < 0.05) in fibroblasts and activation of genes involved in immune responses, migration, and chemotaxis in adipose-derived stem cells derived from surgery donors. Experiments in a model of ear pinna injury in mice indicated that IM moderately promoted tissue repair (8% in BALB/c and 36% in C57BL/6 in comparison to control).

## 1. Introduction

Wound healing complications after trauma, surgery, acute illness, or chronic disease conditions, and subsequent appearance of chronic wounds, affect millions of people worldwide each year. Delayed healing is often caused by dysregulation of the response to wounding resulting from inflammation, angiogenesis, matrix deposition, and/or cell recruitment. The inability to restore the function and architecture of injured tissues is typical of chronic skin wounds resulting from thermal, chemical, or radiation burns of a large area, as well as civilization diseases such as diabetes or atherosclerosis. The need for clinical strategies that might activate natural repair mechanisms leading to scarless wound healing and tissue repair is still growing [1]. One of the possible strategies is pharmacological stimulation of natural regenerative processes in the body. Peptides, which are biocompatible, biodegradable, bioactive, and relatively amenable to large-scale production, are currently gaining interest as potential agents in regenerative medicine. Among many biologically active molecules is the RDKVYR peptide, which is known as thymohexin and marketed as Imunofan (IM). IM is a hydrophilic hexapeptide which was designed based on the sequence at positions 32–37 of the thymopoietin hormone (RKDVYV) (information from the manufacturer’s website). Thymopoietin is produced by the thymus and its function is to increase lymphopoiesis and to selectively induce differentiation of prothymocytes and thymocytes [2]. Studies have shown that certain thymus hormones stimulate regeneration of hematopoietic progenitor cells damaged by ionizing radiation [3].

IM has been reported to demonstrate antioxidative and immunomodulatory properties. The IM peptide, in combination with L-arginine, exerts protective activity in epinephrine- and indomethacin-induced ulcers and gastric lesions in rats [4], where it decreases the activity of inducible nitric oxide synthase (iNOS) as well as the lipoperoxidation process in gastric mucosa. After subcutaneous injection in patients, IM showed immunomodulatory effects, as manifested by decreasing levels of interleukin-6 (IL-6) and tumor necrosis factor (TNF) in response to diphtheria toxoid vaccination [5].

IM causes ultrastructural changes of thyrotropic cells of the adenohypophysis, mainly by increasing their size [6]. It has been proven that application of IM in combination with ozone therapy and surgical treatment can cause improvements in healing processes in orthopedic patients affected by puruloseptic complications [7]. It is also known that stimulation of the immune system by IM, in combination with local electrolyte detoxification, promotes endometrium regeneration in puerperal endometritis. In these studies, migration of lymphoblasts and phagocytes was detected at the endometrial level, thus illustrating endometrium regeneration (Dorina Muntean, PhD thesis “Local electrochemical detoxication and immunomodulation in prophylaxis and treatment of puerperal endometritis”, 2006). In another in vivo experiment, IM displayed a positive effect on cell profiles in the thymus (“Immunomodulator Imunofan affects cell profile of morphofunctional zones of rat thymus and delays its age-related involution“—data published in Russian). The obtained results indicated a delay in thymic involution. The summarized data from the literature show that the IM peptide stimulates immune system activity and it possesses ability to activate the antioxidative system of the human body.

The described above data about pro-regenerative therapies with IM as a supplement and reported immunomodulatory activity of IM, added to the lack of observed side effects [5], encouraged us to investigate whether the peptide has the potential to promote tissue repair and skin wound healing using in vitro and in vivo models. In addition, we performed conformational studies of the IM peptide, in order to search for the structural properties associated with its biological activity.

## 2. Results

In order to evaluate the potential of the peptide RDKVYR (IM) to stimulate tissue repair and skin wound healing, we performed a series of investigations, starting from tests of proliferation and migration through, immunogenicity and transcriptome responses in different cell culture models, to preliminary research using the ear pinna model of tissue repair in mice. Additionally, we performed some physicochemical experiments, which included NMR, CD studies, and stability of IM peptide.

### 2.1. IM Peptide Is Stable in Aqueous Solution, While in Plasma It Is Rapidly Bound by Albumins 

Peptides are susceptible to proteolytic enzymes and are prone to self-degradation [8]. Therefore, the stability of IM in aqueous solution as well as in human plasma was established. After 24 h of incubation of the dissolved peptide in water at 37 °C, there was no reduction of the signal from the IM peptide (Figure S1). It can therefore be concluded that the IM peptide is stable in aqueous solution. On the contrary, after 1 h of incubation at 37 °C, the signal of the IM peptide is no longer seen in plasma (Figure S1). Interestingly enough, the chromatogram of the sample at the time point 0 h, which was a mixture of peptide and plasma without incubation—though it had gone through the whole procedure of sample preparation—showed barely any signal of peptide (Figure S2). During these experiments, albumins were removed from the mixture, before HPLC analysis, using ethanol and centrifugation. If the peptide binds to albumins there is a chance that it will be removed during this procedure. To establish if the peptide binds to albumins we performed affinity tests.

In the supernatant fraction, a *m/z* signal corresponding to an excess of IM peptide was detected (Figure S3A). No signal was observed in the mass spectrum of the last wash fraction, confirming that the excess of IM peptide had been removed and that the column was properly washed out (Figure S3B). The spectrum of the elution fraction showed a *m/z* peak at 836.88 (Figure S3C), which corresponded to the protonated molecule derived from this peptide. It can be concluded that the IM peptide interacted with bovine albumin, since the m/z peak in the elution fraction was consistent with the mass of the peptide.

### 2.2. IM Peptide Adopts a Disordered Structure

As the peptide structure is critical to its biological activity, we performed a series of IM conformational examinations using CD, NMR, and MD techniques.

**Figure 1:**
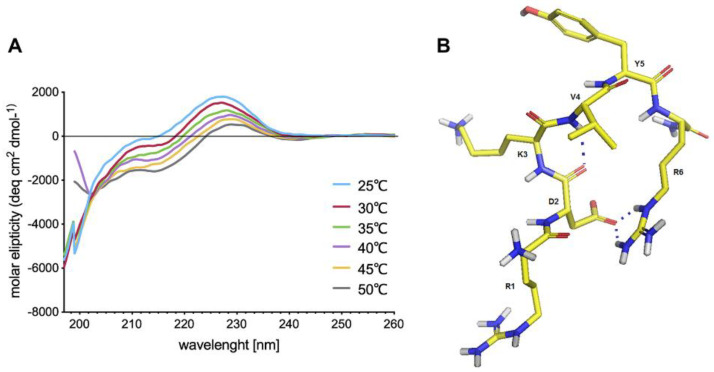
(**A**) CD spectra of Imunofan (IM) peptide in PBS at pH 7.4, over the temperature range 25–50 °C; (**B**) structure of IM obtained after 10 ns of MD simulation in water. The peptide backbone structure is depicted as a stick projection, where the hydrogen bond and salt bridge are marked as dotted lines.

According to CD data, IM adopts a disordered structure regardless of the measurement temperature (Figure 1A). NMR spectra show that the peptide is in a conformational equilibrium between several different conformational states (major and minor signals in the NMR spectra). In these NMR spectra, long-range interactions between Asp2-Arg6 and Val4-Arg6 residues were observed. The spatial structure was determined only for the dominant one and was calculated using the CYANA and AMBER programs with NMR restraints. The results showed that IM adopts a flexible structure in aqueous solution, which was manifested by the presence of minor conformation signals in the NMR spectra (Figure S4 TOCSY). In the final structure, a salt bridge in the major conformation is formed by the oxygen from the side chain of Asp2 and the NH proton from the Arg6 amino acid residue, and there is a hydrogen bond between the main-chain carbonyl oxygen of Asp2 and the NH proton of Val4, which, together, stabilize the turn structure of the whole peptide (Figure 1B). In the structure formed in this manner, the side chains of the Arg1 and Lys3 amino acid residues were strongly exposed to the outside of the molecule, which may affect its biologically properties and its ability to bind to negatively charged surfaces of macromolecules such as proteins or nucleic acids. Knowing from NMR studies that the peptide forms a turn, it might be assumed from looking back at the CD spectra that that turn is indicated by the maximum at 230 nm (Figure 1A).

### 2.3. IM Peptide Is not Cytotoxic to Human Stem Cells and Skin Cell Lines

To assess potential cytotoxicity of IM peptide, we decided to analyze the influence of the peptide on human cells in vitro. A lactate dehydrogenase (LDH) test showed that IM peptide was not cytotoxic to adipose-derived stem cells (ASCs) and human skin cells (Figure S5). In addition, IM peptide was also not toxic to primary neural cells (Figure S6).

### 2.4. IM Peptide Stimulates Proliferation of Human Skin Cells but Does not Stimulate Migration and Chemotaxis of Cells

**Figure 2:**
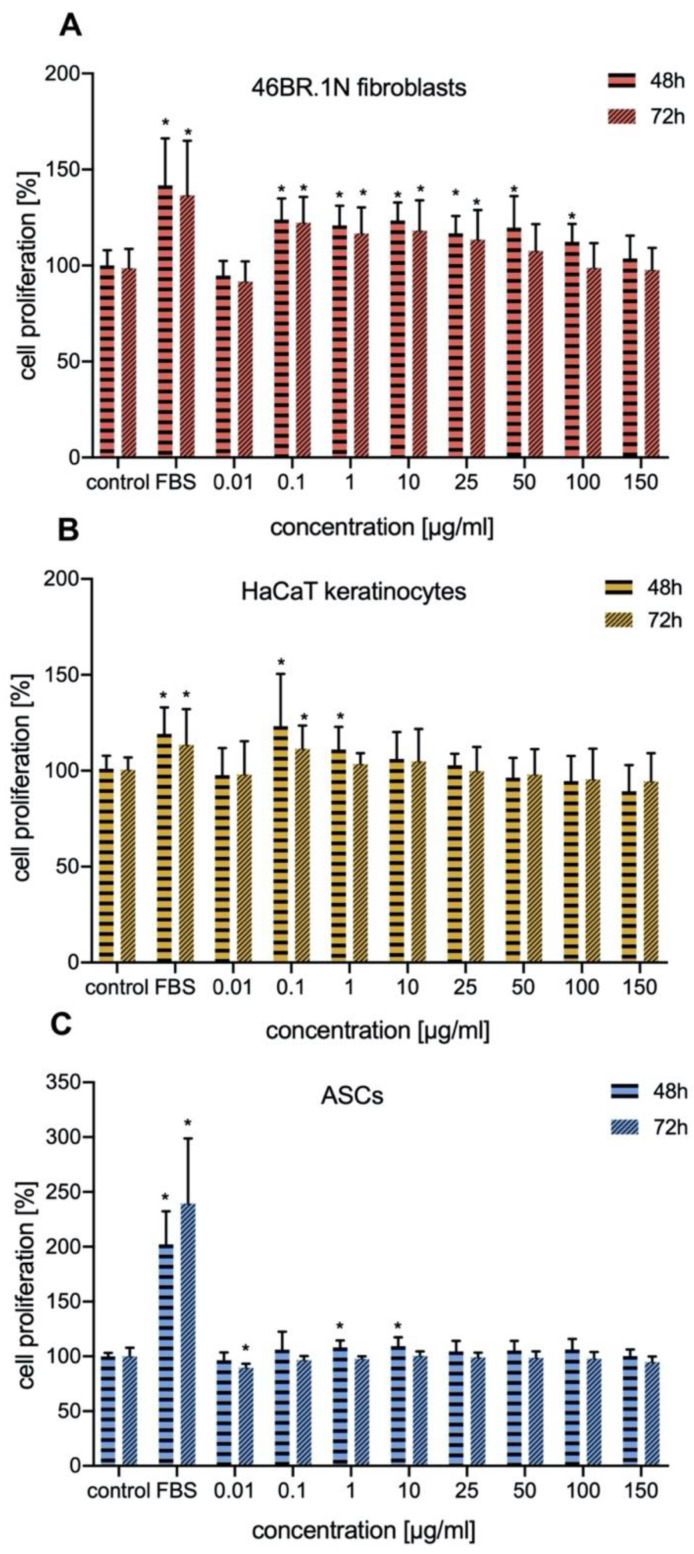
Effect of IM on proliferation of 46BR.1N fibroblasts (**A**) and HaCaT keratinocyte cell lines (**B**) and (**C**) adipose-derived stem cells (ASCs). The graph shows results from 4 independent experiments (4 replicates in each, n = 16). Results are presented as mean with SD. *—statistically significant differences compared to control, Mann–Whitney U test, *p* < 0.05. FBS—positive control-cells grown in medium containing 10% FBS.

Proliferation, migration, and chemotaxis of skin cells play a crucial role in wound healing. Therefore, we decided to investigate the effect of IM on proliferation of 46BR.1N fibroblasts, HaCaT keratinocytes, and human ASCs. The results obtained from XTT analysis of IM’s effect on proliferation of human 46BR.1N fibroblasts (Figure 2A) and HaCaT keratinocytes (Figure 2B) showed statistically significant increases in proliferation of human skin cells and ASCs (Figure 2C). This effect was stronger for the 46BR.1N fibroblast cell line, whose proliferation was increased by 20–40% compared to the control at concentrations of 0.1–100 µg/mL and 0.1–25 µg/mL after 48 h and 72 h of incubation, respectively. For HaCaT keratinocytes a pro-proliferative effect was observed, resulting in a 20–50% increase in cell proliferation only in concentrations of 0.1 and 1 µg/mL and 0.1 µg/mL after 48 h and 72 h, respectively. Moreover, a small but statistically significant pro-proliferative affect was observed for ASCs but only after 48 h of stimulation (Figure 2C). Their proliferation was increased by 10% in concentrations of 1.0 and10 µg/mL. Additionally, IM peptide did not cause inhibition of proliferation of both these tested cell lines and ASCs.

Based on the proliferation analysis, IM concentrations of 0.1 and 1 µg/mL, in which it showed pro-proliferative activity, were chosen for migration analysis. The obtained results of migration demonstrated that IM at the tested concentrations does not stimulate migration of skin cells. For HaCaT keratinocytes there is a slight statistically significant inhibition of migration (5–10% compared to the control) (Figure S7A) and chemotaxis (10–20% with respect to the control) (Figure S7B). Only for ASCs, IM (at 0.1 µg/mL) did show a small, statistically significant promigratory effect (10–15% compared to the control) (Figure S7C).

### 2.5. IM Is Immunologically Safe

**Figure 3:**
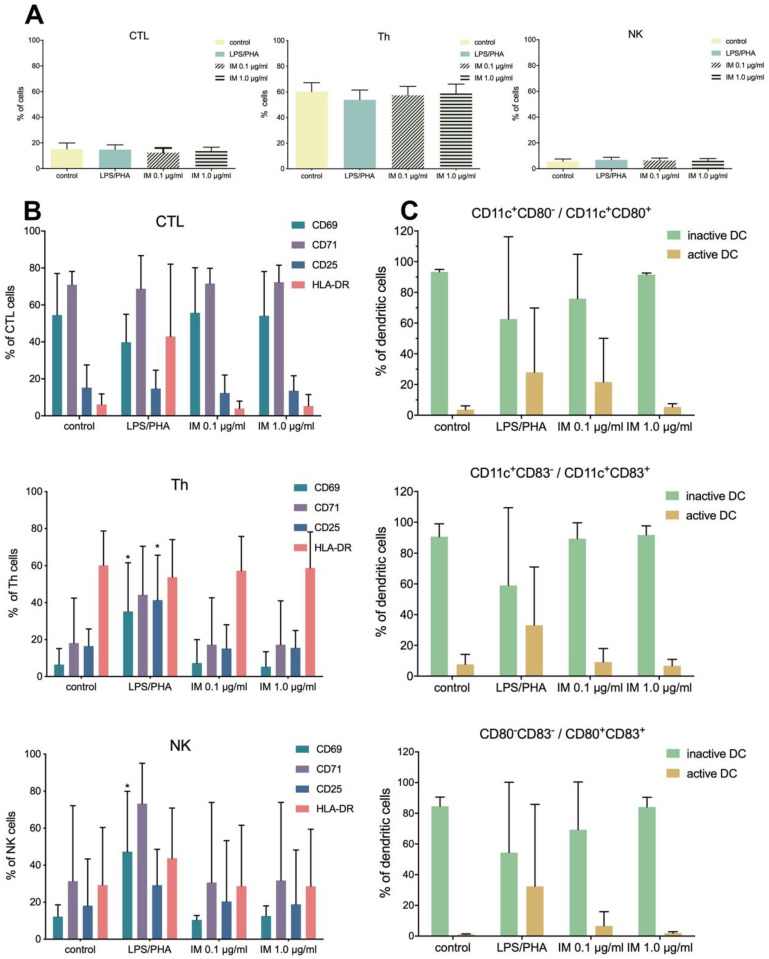
Effect of IM on immune cells activation. (**A**) overall percentage of immune cells subpopulations cytotoxic T lymphocytes (CTL), helper T cells (Th), and natural killer cells (NK), after stimulation with IM (0.1 µg/mL; 1.0 µg/mL). Unstimulated cells were treated as a negative control, while cell cultivated in the presence of LPS/PHA were a positive control. (**B**) percentage of cells expressing activation markers among specific immune cell subpopulations CTL, Th, and NK. (**C**) activation of dendritic cells (DC) subjected to IM, presented as a percentage of cells with active phenotype. The graph shows results from 5 independent experiments. Results are presented as mean with SD. *—statistically significant differences compared to control, Mann–Whitney U test, *p* < 0.05. All data are presented as percentages due to the application of hospital diagnostic laboratory standards for immune cells analyses.

Peptides can induce immune responses and cause allergies, and thus it is important to evaluate the immunogenic and allergic potential of peptides before they are forwarded to expensive clinical trials. Biological tests to evaluate the immunogenic potential of IM were performed on selected immune cell populations (cytotoxic T lymphocytes (CTL), helper T cells (Th), natural killer cells (NK), and dendritic cells (DC)). The obtained results show that IM has no immunogenic properties at both applied concentrations (0.1 and 1.0 µg/mL). The observed activation levels of the analyzed immune cells (CTL, Th, and NK) were not statistically significant and comparable to those of negative control (Figure 3A). Similar observations were made for the levels of activation markers in particular cell subpopulations (Figure 3B). In addition, dendritic cells (Figure 3C) were not activated in the presence of IM (native/inactive phenotype of DC: CD11c+, CD80−, and CD83−; active phenotype: CD11+, CD80+, and CD83+). Therefore, based on the conducted test, the immunological safety of IM was confirmed.

**Figure 4:**
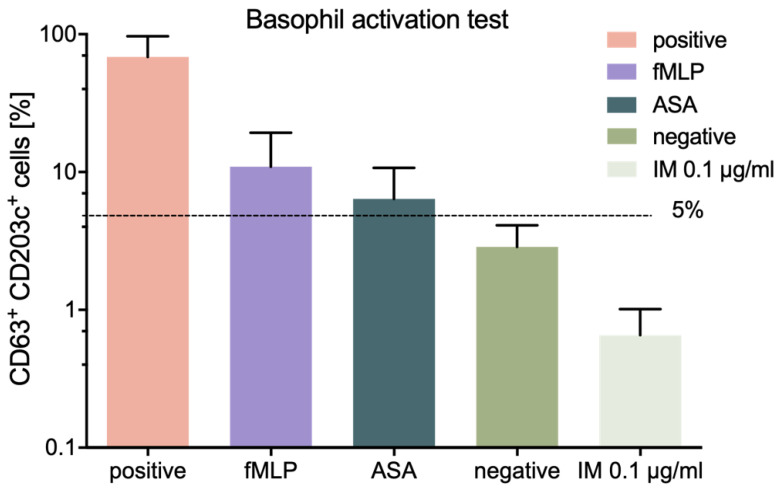
Effect of IM on activation of basophils. The figure shows basophil activation in the presence of: Positive controls, monoclonal antibody FcRI, fMLP, and acetylsalicylic acid (ASA); negative control; and IM peptide (0.1 µg/mL). The graph shows results from 7 independent experiments. Results are presented as mean with SD.

A basophil activation test (BAT) is a refined way for the detection of a hypersensitivity reaction in vitro by flow cytometry. IM (0.1 µg/mL) did not activate basophils and, in all samples challenged with the peptide, the level of degranulated cells remained below 5% (Figure 4), which is a cut-off value above which the test may be considered positive according to the test kit manufacturer. Interestingly, we observed a slight decrease in basophil activation in samples challenged with IM compared to negative control.

### 2.6. IM Does not Induce Significant Changes in The Cytokine Profile in Cultured Cells

The individual traits of various donors may manifest themselves in varied reactions of cells challenged with the same stimuli. Owing to this, it was difficult to distinguish a single pattern of cell responses to IM stimulation. Fibroblast stimulation resulted in a slight, statistically insignificant decrease in the level of IL-8 in all tested samples. The levels of the remaining cytokines/growth factors showed donor-to-donor variability, and no statistically significant differences were found between the stimulated and control cells. This may indicate a direct mechanism of action of IM rather than paracrine regulation by cytokines or growth factors. Protein and cytokine levels in culture supernatants after stimulation with IM are shown in Supplementary Table S1.

### 2.7. Transcriptional Responses to IM Peptide of Genes Controlling Cell Proliferation Potential and DNA Methylation

**Figure 5:**
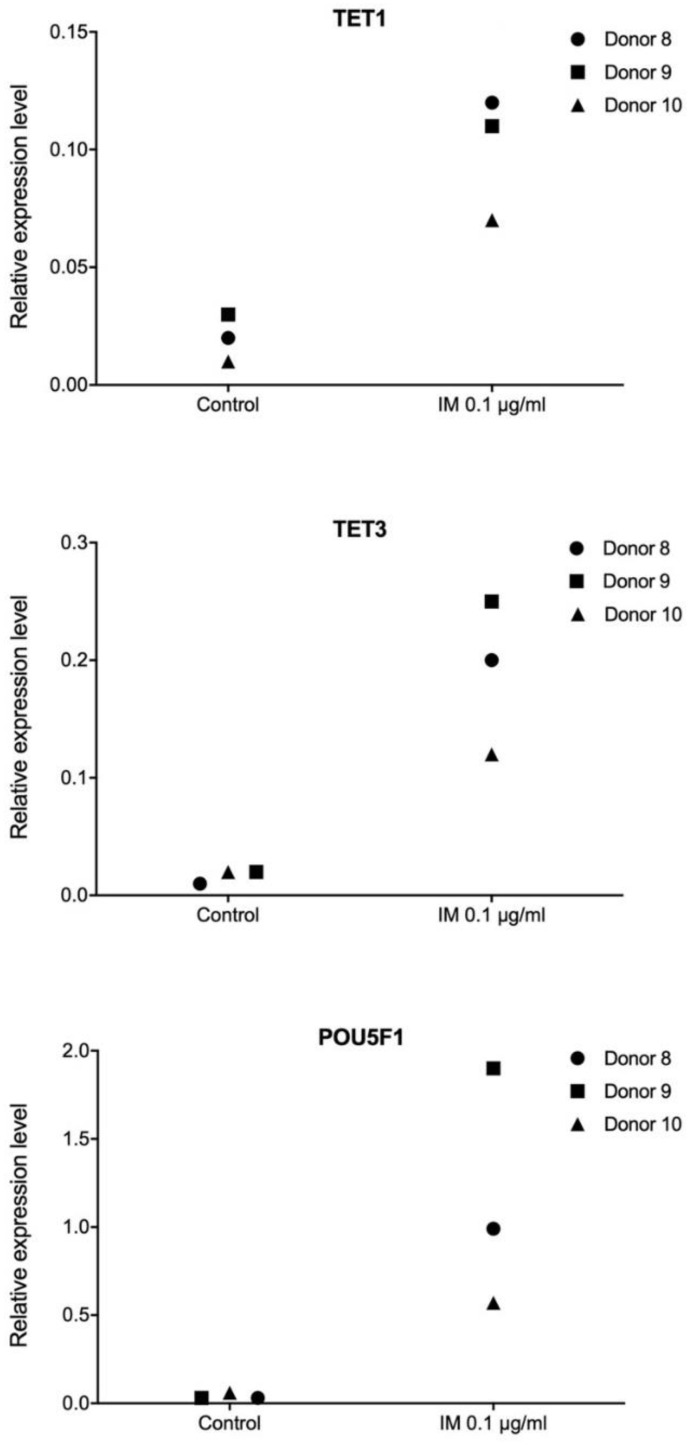
Transcriptional responses of *TET1*, *TET3*, and *POU5F1* to IM at 0.1 µg/mL in primary fibroblast cell cultures. Controls were cultured in the same medium without IM addition. No statistically significant changes were observed (Mann–Whitney U test, *p* < 0.05).

In order to evaluate transcriptional responses to IM stimulation in the context of cell proliferation potential, we determined expression levels for a panel of genes including *CDKN1A*, *MYC*, *KIT*, *POU5F1*, *TGFB3*, and *TP53*, in primary fibroblast (Figure S8a), keratinocyte (Figure S8b), and adipose-derived stem cell cultures (Figure S8c), all derived from donors (surgery patients). Additionally, in primary fibroblast cell cultures, we examined the transcript levels of the following genes which regulate DNA methylation: *DNMT1*, *DNMT3A*, *DNMT3B*, *TET1*, *TET2*, *TET3*, and *TDG* (Figure S9). The analysis revealed no consistent transcriptional pattern changes following IM treatment, however, we did observe some individual responses. Marked increases in expression of POU5F1, which encodes *OCT4*, which itself acts as a decisive factor in inducing and maintaining cell pluripotency, were noted in keratinocyte and adipocyte stem cells from individual donors and there was very strong induction of *POU5F1* in fibroblasts from all three donors in the study (Figure 5). In addition, in fibroblasts, we found high activation of *TET1* and *TET3*, which are key genes responsible for active DNA demethylation (Figure 5).

### 2.8. There Are Transcriptional Responses of ASCs to IM Stimulation

A human ASCs model was selected to study pro-regenerative effect of IM. Ingenuity pathway analysis (IPA) software was applied enabling the analysis of transcript changes in experimental datasets and assignment of them to shifts in upstream regulators and biological downstream effects. The regulatory network (Figure S10A) with the highest consistency score (29.69) was obtained for donor 6 and showed activation of cells, immune response, increase of migration and chemotaxis of cells, and activation of nitric oxide synthesis. The result for donor 11 ASCs stimulated with IM was similar (Figure S10B), though with higher values of *p*-score, while donor 11 showed opposite effects (Figure S10C). Disease and function mode implemented in IPA for ASCs of donor 6 resulted in a list of phenomena that were significantly upregulated in response to IM stimulation (*z*-score ≤ −2, *p*-score  <  0.05) (Table 1).

### 2.9. Subcutaneous Injection of IM Moderately Promotes Tissue Repair in the Mouse Ear Pinna

As the IM peptide showed safety profile and remarkable transcriptomic responses in in vitro tests, we decided to conduct a preliminary experiment in animals. Ear punch wound is a model of tissue repair which has been found useful in testing the pro-regenerative activity of pharmacological agents [9]. Measurements of ear hole closure are made to estimate the regenerative effects. In contrast to the wound healing model of dorsal skin excision, wound contraction does not complicate observations [10].

**Figure 6:**
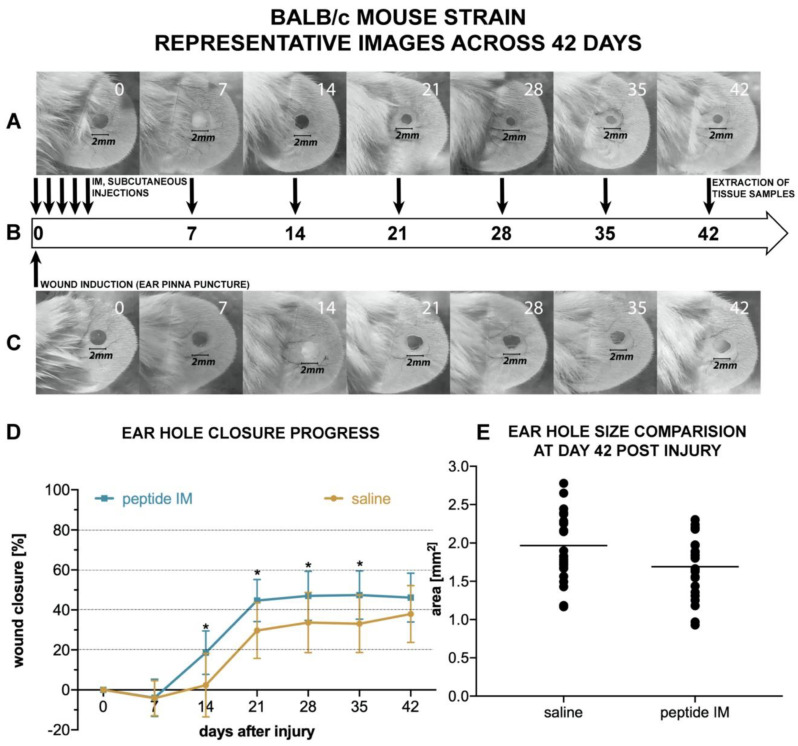
Effect of subcutaneous IM injections on ear punch closure in mice of the BALB/c strain. (**A**) and (**C**) representative photographs depicting the progress of ear hole closure in BALB/c mice, without or with IM stimulation. (**A**) the treatment group (IM injections; n = 12 mice) and (**C**) shows the control group (saline injections; n = 12 mice). (**B**) the timeline of experiments and injection intervals. (**D**) the mean area of BALB/c mice ear holes throughout the experiment; n = 6; error bars represent SD; and statistical significance * means *p* ≤ 0.05. (**E**) the distribution of ear hole area at 42 days post injury in the BALB/c mice strain, where each dot represents one ear hole and the black line represents the mean value.

Experiments were conducted during the course of 42 days (indicated by timeline on Figure 6B) and representative images are shown on Figure 6A,C. Due to limited availability of literature sources, exact concentration of IM peptide for in vivo studies was difficult to establish. References suggests various effective doses for animal studies, ranging from 0.02 µg/gin rats [11] and 0.025 µg/gin mice [12], to as high as 1 mg per mouse (which corresponds to around 40 µg/g [13]). The manufacturer of commercial IM peptide recommends not to exceed a daily dose of 200 µg. Based on the above recommendation, we decided to set the dose at 5 µg/g, which in our case corresponded to a daily dose of around 125 µg of IM peptide per animal. Based on the recommendations from manufacturer of commercial IM, peptide may be delivered every 24–48 h, and the total number of injections should not exceed 20 per course. Furthermore, it is recommended to inject IM more often at the beginning of the study, at then continue throughout the treatment period with maintenance injections. After the first week, we decided to perform additional single injections on the days ear pinnae pictures were also taken (7, 14, 21, 28, 35) to minimize animal distress. Each mouse received 10 injections of IM in total.

Moderate, but statistically significant, ear hole closure was observed in IM-injected BALB/c mice, as shown in Figure 6. Enhanced ear hole closure was recorded in the treatment group, compared to the control group, between days 14 and 42 (Figure 6D). On day 42 post-injury, the mean ear hole area was 1.69 ± 0.38 mm^2^ in the BALB/c treatment group and 1.96 ± 0.48 mm^2^ in the control group, which corresponded to wound closure of 46.2% ± 12.2% and 37.9% ± 14.2%, respectively (Figure 6E). Ear hole closure plateaued around day 28 in both the treatment and control group (Figure 6D). In order to investigate whether the IM-promoted effect on ear punch closure is not specific to the BALB/c strain, we performed analogous experiments in C57BL/6 mice.

**Figure 7:**
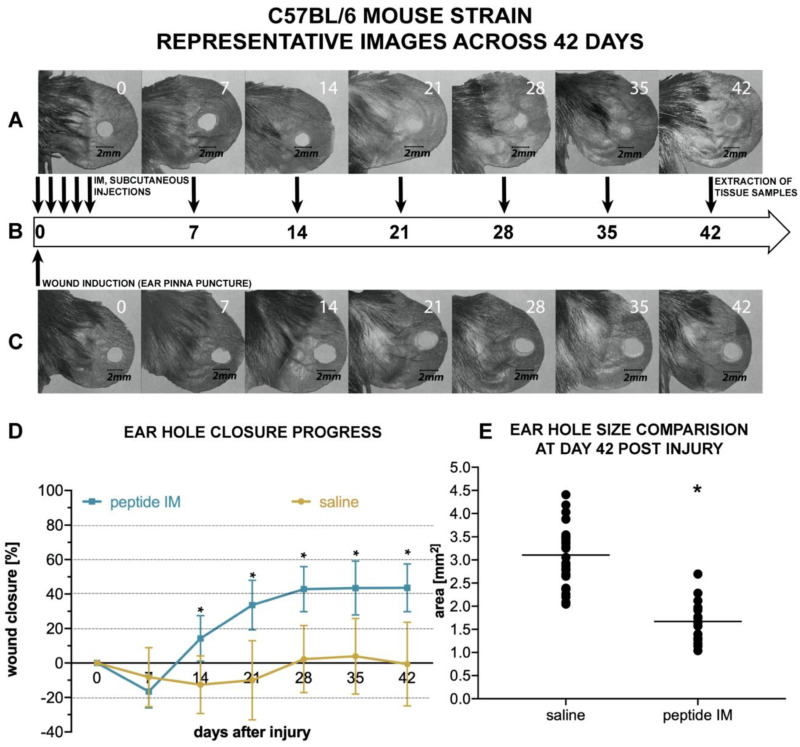
Effect of subcutaneous IM injections on ear punch closure in mice of the C57BL/6 strain. (**A**) and (**C**) representative photographs of healing ear pinnae in C57BL/6 mice injected with saline or IM. (**A**) the treatment group (IM injections; n = 8 mice) and (**C**) the control group (saline injections; n = 18 mice). (**B**) the timeline of experiments and injection intervals. The scale bar is calibrated in mm. (**D**) the mean area of C57BL/6 mice ear holes throughout the experiment; error bars represent SD; and statistical significance * means *p* ≤ 0.05. (**E**) shows the distribution of ear hole area at 42 days post injury in the C57BL/6 mice strain. Each dot represents one ear hole, and the black line represents the mean value.

In C57BL/6 mice strain between days 14 and 42 ear hole area in the treatment group decreased significantly compared to the control group (Figure 7A–C). The mean ear hole area at day 42 post-injury was 1.67 ± 0.45 mm^2^ in the C57BL/6 treatment group and 3.10 ± 0.74 mm^2^ in the control group, which corresponded to 43.6% ± 13.9% and 8.0% ± 36.8% closure, respectively (Figure 7E). In the treatment group, ear hole closure plateaued around day 28 (Figure 7D).

Interestingly, no ear hole closure was observed in C57BL/6 mice injected solely with saline (Figure 7C). Thus, it is worth underlining that BALB/c and C57BL/6 mice responded differently to ear pinna injury, though the effect of IM on ear hole closure was statistically significant in both strains.

**Figure 8:**
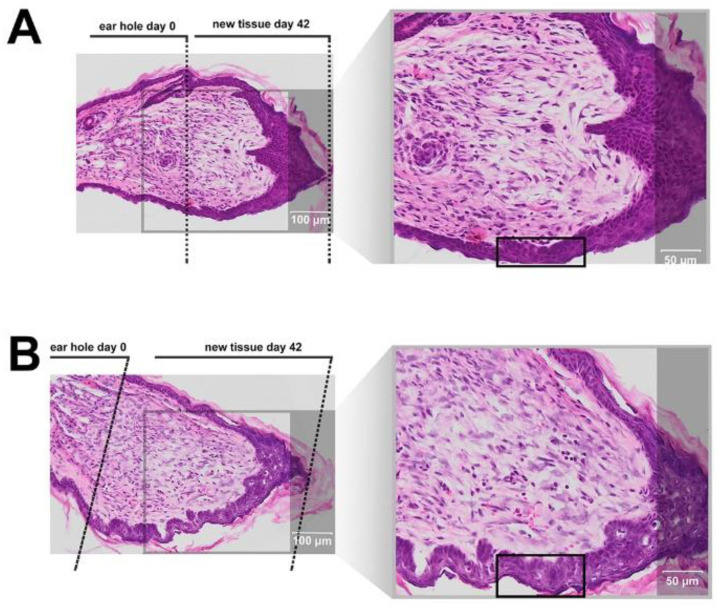
Histological examination of BALB/c mice ear pinna tissue harvested after 42 days of treatment with vehicle only (saline) (**A**) or treatment with IM (**B**). Frames mark enhanced epidermis regeneration. The zone of restoration was calculated by subtracting the radius of ear hole at day 42 from the radius of the initial ear hole.

In order to investigate whether IM affects tissue architecture during ear pinna regeneration, we harvested tissue from mice ears, 42 days after injury (Figure 8). After IM treatment, the epidermis expresses signs of stimulation, which is suggested by a lower level of differentiation related to the ongoing remodeling processes (In Figure 8 enhanced epidermis restoration is marked by black frames). In both IM- and saline-treated wounds epidermal downgrowth was observed, however in the IM treated mice the epidermis showed signs of parakeratosis (the presence of nucleated keratinocytes in the stratum corneum), suggesting accelerated migration of keratinocytes from the basal layer to the stratum corneum. Moreover, the extracellular matrix of the spinous layer in the IM-treated tissue is loose, compared to the control tissue which has more densely packed cells. Overall, the skin in the control mice was dense, basal keratinocytes were flattened, keratohyalin deposits were widely represented, and the epidermis seemed more differentiated. Finally, the central region of IM-treated ear pinnae appears to be more abundant in fibroblasts, in comparison with control ears. However, in general, fibroblast concentration (wound granulation) in both IM-treated and control tissues is similar. In addition, no symptoms of hypertrophic scar formation can be seen in the reconstituted tissues.

## 3. Discussion

Chronic wounds are one of the problems associated with civilization diseases, which creates a demand for finding new therapeutics for promoting skin wound healing. Diabetes, cancers, congenital defects, or postoperative wounds create a mortal threat. Regenerative medicine is trying to develop solutions to these challenges. Therapies are still being sought which could accelerate normal physical responses to injury which might be limited or non-existent in patients with these conditions. Due to the complexity of regeneration processes, multidisciplinary approaches combining cell and molecular biology, genetics, biomaterial sciences, chemistry, bioengineering, and tissue engineering are needed. Peptides appear to display properties that could be useful in promoting skin regeneration processes. They may stimulate fibroblasts to proliferate and to produce elastin and collagen. A good example of a pro-regenerative peptide is a thrombin derived peptide, TP508, also known as Chrysalin (OrthoLogic, Tempe, Arizona—23 amino acid residues), which has been reported to accelerate wound healing [14,15]. Another example is the GHK tripeptide, which participates in the activation of processes related to skin remodeling, and also in activation of fibroblasts leading to the synthesis of collagen and elastin in the human body [16,17]. Further examples of peptides reported to display pro-regenerative properties are Tylotoin [18] and the AH90 peptide [19,20].

In this study a small, hexapeptide, with sequence RDKVYR known as Imunofan, has been tested for potential regenerative properties. IM has been reported to exert an immunomodulatory activity [5] that encouraged us to consider it as an agent for promoting wound healing and tissue repair. For this purpose, we investigated the physicochemical and structural properties of this peptide and characterized its effects on processes related to wound healing, such as proliferation and migration in cultured skin cells. In addition, we performed immunogenicity and cytotoxicity tests on IM and analyzed transcriptional responses to IM stimulation. In order to assess whether IM can promote tissue repair, we examined the results of subcutaneous IM administration in the ear pinna injury model in mice.

Our studies confirm that this peptide is stable in water. We also demonstrated that IM rapidly disappeared in plasma where it was probably bound to albumin, as indicated by affinity tests we carried out. Albumin is a serum protein known to transport large amounts of hormones (e.g., cortisol) [21], drugs (e.g., antibiotics) [22], as well as vitamins [23], fatty acids [24], and lipids [25]. However, the fact that IM is rapidly bound by albumin, is not a proof that IM can be transported to the wound area. To verify this, additional experiments are needed beyond the scope of this manuscript.

Cell receptors for IM have not been identified yet but examination of its spatial structure may provide clues about the nature of possible IM interactions with target proteins. Despite IM being a relatively short peptide shows a tendency to adopt a bent structure stabilized by a strong salt bridge and a hydrogen bond. Nevertheless, IM shows high conformational flexibility, and thus it may be adaptable to multiple different receptors hence spatial structure yields no clues.

In our previous work [26], we optimized a procedure to use fibroblasts, keratinocytes, and ASCs as models in skin regeneration studies. We used serum free culture media, because addition of FBS, which contains various growth factors, which stimulate cell proliferation or migration, could mask IM’s effect. Moreover, IM could be captured by albumins present in FBS, which probably would minimize its effect. We found that IM is not cytotoxic to human skin cells (fibroblasts and keratinocytes). It also does not influence the viability of neural cells (Supplementary Materials Figure S6) and proliferation of two different breast cancer cell lines (see Supplementary Materials Figures S11 and S12). The lack of cytotoxicity of IM to different cells is a great asset, indicating that it can be used without the risk of damaging cells and tissues. The low cytotoxicity of IM demonstrated in this research corroborates the results of other studies [4,27].

In our work we investigated the effect of IM on proliferation of human fibroblasts and keratinocytes. The proliferation of these cells is crucial for proper skin regeneration processes. Our studies indicated that IM stimulated proliferation of human skin cells (fibroblasts and keratinocytes), mainly in concentrations of 0.1–25 μg/mL, with similar effect on both types of cells. It also slightly stimulate proliferation of human ASCs. Similar studies have been conducted for TP5, a peptide which is very similar to IM. TP5 has no inhibitory effect on proliferation of healthy human peripheral lymphocytes and peripheral blood mononuclear cells (PBMCs) [28,29], and at the same time it induces proliferation of spleen cells [30].

Migration and chemotaxis of fibroblasts and keratinocytes, as well as paracrine factors are essential for skin wound healing. In the present study, IM was not found to stimulate migration and chemotaxis of skin cells at any of the tested concentrations, which indicated that its biological actions are not related to these activities. Cells undergoing intensified processes of proliferation usually have limited migration and chemotactic properties [31].

Peptides play an important role in immunology, often through being epitopes for adaptive immune systems [32,33]. Besides, they take part in mobilizing immune cells. For example, cationic host defense peptides, which possess antimicrobial properties, have the ability to control infections and inflammation because of their influence on innate and adaptive immunity [34]. Unfortunately, application of synthetic peptides can lead to unwanted immune activation or allergies and immunogenicity or immune-related adverse effects can limit the effectiveness of peptide-based therapies [35,36,37]. IM has been reported to be a compound with in vivo immunoregulatory properties, for example it has been reported to increase the production of IgM, IgG, and IgA in human lymphocyte cultures and to suppress the synthesis of IgE. However, IM has been found to induce the production of immunoglobulins following stimulation with pokeweed mitogen but no influence has been observed without pre-treatment of lymphocytes with the mitogen [38]. Our studies were conducted in vitro on isolated PBMCs and indicated that IM did not activate the immune system (no activation of CTL, Th, and NK lymphocytes), thus suggesting IM is safe for use.

We also assessed the allergic potential of IM peptide, using an in vitro method, namely the BAT described previously [26]. The conducted tests showed low allergic potential of IM peptide, thus confirming the immunological safety of this peptide.

In our research, we also examined the effect of IM on the secretion of specific growth factors and cytokines by fibroblasts, keratinocytes, and ASCs. For this purpose, we applied Luminex technology (Luminex xMAP), which is a trusted, widely used platform for biomarker screening and protein analysis. Our results showed that IM exerted little impact on the secretion pattern of cells.

Pluripotency is defined as the ability of a cell to differentiate into any type of somatic cell, apart from trophoblasts. Establishment and maintenance of a pluripotent state depends on a group of transcription factors. Therefore, in our work, we performed transcriptome profiling in primary human fibroblasts, keratinocytes, and ASCs, in order to observe extensive alterations following stimulation with IM peptide. Transcriptional responses to IM of genes controlling cell proliferation potential and DNA methylation were evaluated. The analysis revealed no consistent transcriptional pattern changes following IM treatment, however, we did observe some individual responses. We found very strong induction of *POU5F1* encoding *OCT4,* a key pluripotency factor, as well as of the *TET1* and *TET3* genes involved in active DNA demethylation. A recent study suggests that *TET* enzymes play roles in controlling the expression of genes regulating regenerative processes in the skin [39]. *OCT4* is the main factor responsible for pluripotency induction [40], and it retains high activity in pluripotent cells and is quenched after differentiation [41]. Interestingly, as described by Costa et al. [42], *TET* dioxygenases and *OCT4* are likely to induce local transcriptional changes in shared target genes that regulate pluripotency.

Transcriptome profiling is a useful tool for evaluation of potential drugs. It provides an insight into the activity of investigated molecules. High-throughput next generation sequencing of RNA which can reveal mechanisms of action of tested pharmacological agents, thanks to its potential to characterize and quantify genome-wide gene expression [43]. Utilization of this technology for investigating the effect of PDGF2 peptide administration on skin cells is presented in our previous research [26]. In this work, we also performed RNA sequencing analysis for ASCs stimulated with IM. Although the response to IM varied between individual donors, we were able to observe the activation of processes related to wound healing: Activation of cells, proliferation and cell cycle progression, a pathway for synthesis of nitric oxide, and slight anti-inflammatory activity.

There are few animal models used for assessing the potential pro-regenerative activity of chemical compounds. The ear punch wound model in mice, though simple to perform, is a powerful model of complex tissue regeneration in mammals [9]. The experiment involves making a 2 mm hole in the center of ear pinna in normal laboratory mice, such as BALB/c ad C57BL/6J. Quantitation of ear pinna hole closure in response to pharmacological treatment is used to assess the pro-regenerative activities of tested chemical compounds. Histological examination is also performed to evaluate if tissue architecture is restored within the re-grown region, which indicates a true regenerative response. In the presented research, we carried out tests of IM healing effects using this model. While limited ear hole closure was determined in mice administered physiological saline (38% in BALB/c and 8% in C57BL/6), restoration of lost tissue was observed in the IM-treated animals (46% in BALB/c and 44% in C57BL/6) 42 days after injury. Subcutaneous injection of IM activated moderate tissue repair in both mouse strains, however a much stronger effect was observed in C57BL/6 mice. Histological examination showed that the restoration in ear pinnae involved not only the formation of connective tissue but also the presence of numerous fibroblasts and epithelial cells. However, the presented work did not fully investigate the mechanism of IM action. Hence, further studies are required to determine molecular and cellular mechanism of IM action. A more detailed analysis of IM effect on immune cells is also necessary.

## 4. Materials and Methods

### 4.1. Peptide Synthesis and Purification

The IM peptide (H-Arg-Asp-Lys-Val-Tyr-Arg-OH) was synthesized by the standard solid phase method using *N*-Fmoc protected amino acids in a Liberty BlueTM automated microwave synthesizer (CEM Corporation, Matthews, NC, USA). Cl-TCP(Cl) ProTide (CEM Corporation, Matthews, NC, USA) resin was used as a solid support with a capacity of 0.4 mmol/g. During the synthesis, a 20% piperidine solution in DMF was used as a remover of the Fmoc amino group, a 0.5 M DIC solution in DMF was used as the coupling reagent, a 1 M solution of Oxyma pure was used as a suppressor of racemization, and 0.1 M DIPEA in DMF was used as a scavenger of free radicals. The peptide was cleaved from the solid support, along with removal of protecting groups from the amino acid side chains, using the mixture TFA/phenol/TIPSI/H_2_O (88:5:5:2 v/v/v/v). The IM peptide was purified by preparative high-performance liquid chromatography on a reversed phase (RP-HPLC), at room temperature, using an appropriate water/acetonitrile gradient with TFA. The crude product of the synthesis was purified using a Jupiter^®^ Proteo C12 semipreparative column (Phenomenex, Torrance, CA, USA) with dimensions 21.2 mm by 250 mm, 4 μm, 90 Å. The pure product was analyzed using analytical RP-HPLC and mass spectrometry (BIFLEX III MALDI-TOF, Bruker, Germany, and ESI LCMS IT TOF, Shimadzu, Japan) (see Supplementary Data Figure S13). The purified peptide was subjected to exchange of the trifluoroacetate counter ions to acetate using 0.1 M aqueous ammonium acetate.

### 4.2. Stability in Water and Plasma

A stock of IM peptide in sterile water (1 mg/mL) was prepared and divided into 200 µL portions. The sample tubes were stored in an incubator at 37 °C and analysis was performed at various time points (0, 1, 2, 3, 6, and 24 h). The analyses were performed by HPLC using a Luna C18(2) (5 µm, 100 Å 4.6 by 250 mm) column (Phenomenex, Torrance, CA, USA) and monitored by a PDA detector. Then, 0.01% TFA in H_2_O (A) and 0.01% TFA in 80% ACN in water (B) were used as solvents. A linear gradient of 5–100% B over 60 min and a flow rate of 1 mL/min was applied.

A stability test in plasma was performed as previously described [44]. Blood was collected from healthy anonymous donors and EDTA was then used as an anticoagulant. The plasma was aliquoted and incubated with IM peptide for 1, 2, 3, 6, and 24 h at 37 °C, at a final concentration of 300 µM. The analyses were performed by HPLC applying the conditions specified above.

For calculations, peak areas were compared to a calibration curve calculated by LC solution Software (Shimadzu, Kyoto, Japan).

### 4.3. CD and NMR Studies

CD studies of IM were performed in PBS (pH 7.4). The CD spectra were recorded for 0.25 mg/mL peptide, over the range 185–260 nm, with a Jasco J-815 spectropolarimeter (Jasco, Victoria, BC, Canada). For IM peptide, the CD spectra were recorded three times, over a temperature range of 25–50 °C and using a 1-mm cell, and depicted as the mean residue molar ellipticity (MRME, deg × cm^2^ × dmol^−1^) against the wavelength λ (nm).

NMR experiments were performed in water (90%:10% H_2_O:D_2_O). The peptide concentration was 4.0 mM. All NMR datasets were acquired at 303 K on a Bruker Avance III NMR System 700 NMR spectrometer (Brucker, Billerica, MA, USA). Proton chemical shifts (ppm) of IM peptide are listed in Supplementary Table S2. Sequential backbone resonance assignments were obtained according to a standard procedure based on analysis of the 2D NMR spectra [45]. In our study, the analyzed NMR datasets included the following: (i) double-quantum filtered correlation spectroscopy (DQF-COSY) [46]; (ii) total chemical shift correlation spectroscopy (TOCSY) [47], acquired at a mixing time of 80 ms; (iii) nuclear Overhauser effect spectroscopy (NOESY) [48], recorded with mixing times of 200 ms; and (iv) rotating-frame Overhauser enhancement spectroscopy (ROESY) [49], collected with mixing times of 200 ms. The acquired datasets were processed with NMRPipe software [50] and analyzed with the Sparky [51] program.

### 4.4. Structure Calculations

The interproton distances were calculated on the basis of the NOE intensities extracted from the NOESY spectra at a mixing time of 200 ms. Here, 98 distance restraints were provided for IM. The initial structure of the peptide was calculated by a simulated annealing algorithm, which included 10,000 steps of MD in the torsion angle space (CYANA software, Frankfurt, Germany) [52]. As a result, the calculation protocol initialized with 200 structures created randomly chosen torsion angles. The 3D structures of IM, evaluated with CYANA, were used as the starting structure in the MD calculations in water. The entire system was subjected to energy minimization (steepest descent method). The system was then subjected to NTP MD for 10 ns at 305 K, with time steps of 2 fs. During the MD stimulation, a non-bonded cutoff of 9 Å was chosen. The MD studies in water were performed with AMBER software [53], using a protocol including time-averaged NMR restraints (TAV) derived from NMR spectroscopy, with the force constant for interproton connectivity equal to 50 kcal/(mol × Å^2^). No torsion angle restraints were used in the calculations. The geometry of the peptide groups was kept all trans, with the force constant of f = 50 kcal/mol × rad^2^, according to the NMR data. The coordinates were collected every 2,000 steps. The shape of the IM structure was evaluated using the PyMOL program [54].

### 4.5. Affinity Test

Immobilization of protein in a microcolumn, and affinity tests, were performed as described previously by Spodzieja et al. [55]. The IM peptide was added onto an albumin-Sepharose column equilibrated in ammonium hydrogen carbonate, pH 7.4, and incubated for 2 h at 25 °C with gentle shaking. After 2 h, the column was washed with 100 mL of ammonium hydrogen carbonate, collecting the first and last milliliter of the solution (supernatant and last wash fraction). The albumin-peptide complex was dissociated by incubation for 15 min in 0.1% TFA, with gentle shaking. The procedure was repeated twice, and the elution fraction was collected each time. All fractions were lyophilized and analyzed by mass spectrometry (ESI-IT-TOF, Kyoto, Shimadzu Japan).

### 4.6. Primary Cell Isolation

Human skin and subcutaneous adipose tissue were sampled from patients of the Plastic Surgery Clinic or Oncologic Surgery Clinic in the Medical University of Gdańsk. The procedure was approved by the Independent Bioethics Commission for Research of the Medical University of Gdańsk (NKBBN/387/2014). Tissue samples for cell isolation were obtained from adult donors (Table S3). Unchanged skin was removed routinely during surgery, e.g., abdominal surgery or mastectomy and adipose tissue was removed with skin fragments or by liposuction. Donors with other medical conditions that could affect the results of tests, e.g., active infections and autoimmune diseases (e.g., scleroderma, lupus, MS), HBV, HCV, HIV carriers, patients with active allergy, diabetes or atherosclerosis, and advanced cancer as well as subjected to neoadjuvant treatment (chemotherapy, radiotherapy, and/or immunotherapy) were excluded from the study. All tissue donors were informed about the assumptions of the project and signed an informed consent. Isolation of adipose-derived stem cells was based on a standard protocol, followed by enzymatic digestion and erythrocyte lysis [56,57]. Confirmation of “stemness” of these cells was performed by flow cytometry and analysis of differentiation potential into adipocytes, osteocytes, and chondrocytes (Figure S14), as previously described by Mieczkowska et al. [56], and the protocol is summarized in the Supplementary file. Primary epidermal cells were isolated with the protocol described earlier by Langa et al. [58] and fibroblasts by culture of explants. To allow cells to grow out from the tissue samples, human dermis was placed in six-well plates containing Dulbecco’s modified Eagle’s medium high glucose (4500 mg/L) medium supplemented with 10% of fetal bovine serum (FBS, Sigma-Aldrich, St. Louis, MO, USA), 100 U/mL of penicillin, and 100 μg/mL streptomycin (Sigma-Aldrich, MO, USA), then placed under standard conditions in a humidified atmosphere with 5% CO_2_ at 37 °C. The medium was changed every 2–3 days.

### 4.7. Cell Culture Conditions

Five types of cells were used in our study: Immortalized human HaCaT keratinocytes (DKFZ Heidelberg, Germany), human dermal fibroblasts of cell line 46BR.1N, human primary fibroblasts and keratinocytes isolated from skin samples, and ASCs. The fibroblast cell line was originally derived from the skin of an anonymous individual and was transformed with the pSV3neo plasmid [59]. HaCaT keratinocytes were immortalized cells derived from normal epidermal keratinocytes. They maintain most of the normal keratinocyte functions, including differentiation potential and response to different stimuli [60,61]. HaCaT, 46BR.1N cells, and human primary fibroblasts were grown in a DMEM medium (Sigma–Aldrich, St. Louis, MO, USA; including 4500 mg/L of glucose, 584 mg/l of l-glutamine, sodium pyruvate, and sodium bicarbonate) supplemented with 10% FBS and 100 units/mL of penicillin and 100 μg/mL of streptomycin (Sigma–Aldrich, St. Louis, MO, USA). Human primary keratinocytes were grown in Keratinocyte Growth Medium (cat. CC-3103, Lonza-Clonetics, Basel, Switzerland) supplemented with epidermal growth factor, hydrocortisone, transferrin, epinephrine, insulin, and gentamycin (cat. CC-4152, Lonza-Clonetics). ASCs were grown in DMEM (Sigma–Aldrich, St. Louis, MO, USA; with 1000 mg/L of glucose, 584 mg/L of l-glutamine, sodium pyruvate, and sodium bicarbonate) supplemented with 10% FBS and 100 units/mL of penicillin and 100 μg/mL of streptomycin (Sigma–Aldrich, St. Louis, MO, USA). The cells were routinely cultured under a humidified atmosphere, with 5% CO_2_ and at 37 °C, in culture flasks (growth surface area 25 cm^2^).

### 4.8. Peptide Cytotoxicity Assay

Cell death was quantified by measurement of lactate dehydrogenase activity in cell culture supernatants (Takara, Japan, cat. No. MK401), following the manufacturer’s instructions. Cells were seeded into 96-well plates (BD, cat.no. 353872), at a density of 5000 cells per well, in medium (DMEM) supplemented with 10% FBS. After 24 h, the medium was changed to serum-free medium containing appropriate concentrations of IM peptide. After 48 h of stimulation supernatants were collected for LDH analysis. Cell death was normalized with respect to untreated control (without IM). Non-IM treated cells were used as negative control (0%) and Triton-X 100 (1%) was used as the positive control for maximum LDH release (maximum cytotoxicity, 100%) [26,62]. The assay was performed to IM in concentrations of 50–150 µg/mL. IM did not show signs of toxicity in lower concentrations, so it was not necessary to perform LDH analysis for other concentrations.

### 4.9. Skin Cell and ASCs Proliferation Assay

XTT assay, which is widely used in the measurement of the effect of bioactive compounds (e.g., cytokines, growth factors, and peptides) on cell proliferation was used for the evaluation of IM effect on skin cells and ASCs proliferation [63,64]. Cells were seeded at a density of 5000 cells per well into 96-well plates (BD, cat.no. 353872), in a medium supplemented with 10% of FBS, which after 24 h was exchanged for a serum-free DMEM medium containing appropriate concentrations of the IM peptide. IM solutions used in the experiments were prepared with double distilled water under sterile conditions. The cells were incubated with the peptide for 48 h or 72 h, then XTT reagent was added and the plates were incubated at 37 °C for 4 h in the presence of 5% CO_2,_ following manufacturer’s instructions (Sigma-Aldrich, St. Louis, MO, USA). Plates were then read using a standard plate reader at OD 490 nm. Cell proliferation was normalized with respect to an untreated control (100%) [26].

### 4.10. Migration and Chemotaxis Assay

The effect of IM on cell migration after 24 h was determined using ibidi culture inserts (ibidi, Cat. No. 81176, Gräfelfing, Germany). Cells were seeded in culture inserts, at a density of 30,000 per well, in appropriate medium supplemented with 10% FBS, which was changed to serum-free DMEM after 24 h. Mitomycin C (5 μg/mL) was added for 2 h to block cell proliferation. The medium was then changed, again, and cells were stimulated with an appropriate concentration of IM. After 24 h the cells were fixed with 3.7% paraformaldehyde, stained with 0.05% crystal violet, and the effect was measured with a phase-contrast fluorescent microscope (Zeiss, Oberkochen, Germany) and the surface areas were counted with Imaging Software NIS-Element Basic Research program [26,62].

The effect of IM on cell chemotaxis was assessed with ThinCert cell culture inserts (8 μm, Greiner Bio-One, Kremsmünster, Austria). Cells were starved overnight in serum-free high-glucose DMEM and then seeded on inserts placed in a 24-well plate, at a density of 100,000 cells, in serum-free medium. Culture medium with appropriate concentrations of IM was then added to the wells of the plate and incubated for 24 h. The cells were stained with 8 μM Calcein-AM (BD Pharmingen), then migratory cells were detached with typsin-EDTA (Sigma-Aldrich, St. Louis, MO, USA). Detached cells were transferred to another plate and read with a fluorescence plate reader at an excitation wavelength of 485 nm and an emission wavelength of 520 nm [26].

### 4.11. Immunological Studies

Immunological tests were conducted on human peripheral blood mononuclear cells, isolated from “buffy coats” using a ficoll density gradient (Histopaque, Sigma-Aldrich, St. Louis, MO, USA). Buffy coats for were obtained from blood of healthy anonymous donors of the Regional Center of Blood Donation and Blood Therapy in Gdansk, Poland. After washing in PBS, erythrocyte lysis, and cell counting (cell counter, Bio-Rad), the PBMCs were seeded onto a 24-well culture plate at a density of 10^6^ cells/1 mL of RPMI 1640 (Penicillin/Streptomycin, 10% FBS) per well. After cell resting for 24 h, cells were stimulated with IM peptide (0.1 µg/mL and 1.0 µg/mL) for the next 48 h under appropriate conditions (37 °C, 5% CO_2_). The untreated cells were used as a negative control, whereas cells stimulated with LPS (1 µg/mL) and PHA (2.5 µg/mL) were a positive control. After incubation, the PBMCs were collected, washed with PBS, counted and prepared for flow cytometry analysis. 10^4^ cells/100 µl were stained with monoclonal, fluorochrome-conjugated antibodies (anti-CD3, -CD4, -CD8, -CD16, -CD56, -CD25, -CD69, -CD71, -HLA-DR, -CD11c, -CD80, and -CD83) and incubated for 30 min in RT in the dark. Stained cells were analyzed using a flow cytometer (LSRFortessa, BD, USA) [26,62]. All data are presented as percentages due to the application of hospital diagnostic laboratory standards for immune cells analyses.

### 4.12. Basophil Activation Test

The commercially available test Flow CAST^®^ highsens (Bühlmann Laboratories, Schönenbuch, Switzerland) was used to perform BAT test on blood samples collected from healthy volunteers, who were not suffering from allergy, within 24 h of collection, according to the manufacturer’s protocol. Then, 100 µL of blood was incubated and IM peptide was added to the wells at a final concentration of 0.1 µg/mL. The cells were then stained with fluorochrome-stained monoclonal antibodies (anti-CD63, -CD203c, -CCR3) and incubated for 15 min at 37 °C. Then, erythrocyte lysis was performed, the cells were washed and analyzed by flow cytometry (BD FACSCanto II, San Jose, CA, USA). Each sample was accompanied by its own negative and positive control (anti-FcεRI mAb and fMLP) [62,65].

### 4.13. Analysis of Protein and Cytokine Levels in Culture Supernatants

We used Luminex^®^ xMAP^®^ technology for analysis of proteins and cytokines in supernatants collected from primary fibroblasts or keratinocytes and cell cultures of ASCs stimulated with IM peptide at 0.1 µg/mL concentration. This method allows multiplex detection of proteins in single biological samples. For analysis of ASC supernatants we used Human Adipocyte Magnetic Bead Panel (Merck Millipore, Burlington, MA, USA) which enables simultaneous quantification of the following: Adiponectin, HGF, IL-1β, IL-6, IL-8, leptin, MCP-1, NGF, PAI-1 (total), resistin, and TNFα. For supernatants of fibroblasts, keratinocytes, and ASCs we assessed concentrations of 12 human cytokines and growth factors (angiopoietin-2, BMP-9, EGF, endoglin, endothelin-1, FGF-1 (acidic FGF), FGF-2 (basic FGF), follistatin, G-CSF, HB-EGF, HGF, IL-8, leptin, PLGF, VEGF-A, VEGF-C and VEGF-D) with Human Angiogenesis/Growth Factor Magnetic Bead Panel 1 (Merck Millipore, Burlington, MA, USA). The analysis was conducted according to the manufacturer’s instructions with a protocol described before by Deptula et al. [26]. Analysis was performed with a Luminex MAGPIX^®^ Analyzer (Merck Millipore, Burlington, MA, USA) and data was analyzed in xPONENT 4.2 software. Results are presented as the concentration of cytokines in units of pg per 1 mL.

### 4.14. Cells Stimulation for Transcriptome Analysis, for Transcript Level Analysis and for Luminex Xmap Analyses

Cell stimulation was performed as previously described [26]. Primary fibroblasts after the third passage and ASCs after the second passage were cultured in appropriate DMEM medium supplemented with 10% FBS for 24 h. Then, the medium was changed for DMEM with 5% FBS and 24-h incubation was performed. After another medium change for serum-free, cells were stimulated with IM peptide (0.1 µg/mL) for 48 h. Cell cultures deprived of FBS for 48 h were used as a control. After stimulation, the cells were trypsinized, collected, and centrifuged, and the pellet was kept frozen (−80 °C) for transcriptome analysis and transcript level analysis.

Human epidermal cells were cultured in a 25 cm^2^ t-flask in keratinocyte growth medium (KGM, Lonza, Basel, Switzerland) supplemented with epidermal growth factor, hydrocortisone, transferrin, epinephrine, insulin, gentamycin, and 5% FBS for 24h. Then, they were grown in serum-free KGM for another 2–5 days. Next, the medium was exchanged for keratinocyte basal medium (KBM, Lonza, Basel, Switzerland) and the cells were stimulated with IM peptide (0.1 or 1.0 µg/mL) for 48 h. The cell pellets were collected and kept frozen at −80 °C until RNA isolation for transcriptome analysis and transcript level analysis.

Cell culture supernatants of ASCs, human primary fibroblasts and human epidermal cells were collected and stored frozen at for further Luminex xMAP analyses.

### 4.15. Transcript Levels

RNA was extracted using an RNeasy Mini kit (Qiagen, Cat. No. 74104, Hilden, Germany). RNA quality and concentrations were examined with a NanoDrop 2000 spectrophotometer. cDNA synthesis was performed in a reaction mixture containing 200 ng of RNA, 100 pmoles of oligo dT20, 4 µl of 5× reaction buffer (250 mM Tris-HCl, 375 mM KCl, 15 mM MgCl_2_, and 50 mM DTT), and 200 units of Maxima Reverse Transcriptase (ThermoScientific Bio. Cat. No. EP0742, Waltham, MA USA), at a final volume of 20 µl. PCR reactions were carried out on a LightCycler LC96 device (Roche, Basel, Switzerland), at a final volume of 10 µl containing 5 µl of FastStart Essential DNA Green Master (Roche, Cat. No. 06402712001, Basel, Switzerland), 1 µl of cDNA, and 0.25 µl each of forward and reverse primer (10 µM). The primer sequences are listed in Supplementary Table S4. The transcript levels were determined using the 2^−ΔCt^ method relative to *Actb* and *Tbp* [9,26].

### 4.16. RNA Isolation and Quality Assessment for RNAseq

For three plastic surgery donors (donor 6, donor 11, and donor 12) ASCs (3–4.5 × 10^5^ cells) after passage P2, namely cultures stimulated for 48 h with 0.1 µg/mL IM, or control cultured with FBS-deprived medium, were trypsinized, washed, and snap frozen for transcriptome analysis (see Supplementary Table S5 for clinical information). The pellets were stored at −80 °C prior to RNA isolation. RNA was isolated using a miRNeasy Mini Kit (Qiagen, Hilden, Germany) with two modifications of the original protocol: (i) chloroform was substituted with 1-bromo-3-chloropropane to prevent foaming and emulsification, and (ii) the elution was done with 40 µl of water followed by another elution with the entire volume of the original eluate. The quality and concentration of RNA was assessed using a Bioanalyzer 2100 Instrument and RNA 6000 Nano Kit (Agilent, Waldbronn, Germany)**.** The average RNA yield was 2 µg. Samples with RIN (RNA Integrity Number) >8.0 were used for further transcriptome analysis [26,56].

### 4.17. RNA Sequencing and Bioinformatic Analysis

A ribosome reduction kit (Arraystar Inc, Rockville, MD, USA) and SureSelect Strand Specific mRNA library kit (Agilent, Santa Clara, CA, USA) were used for next generation sequencing (NGS) library preparation according to standard procedures. The cDNA libraries were quantitated using qPCR in a Roche LightCycler 480 with the Kapa Library Quantification Kit for Illumina platforms (Kapa Biosystems, Woburn, MA, USA). Onboard cluster generation and 50 nt paired end RNA sequencing were performed on an Illumina HiSeq2500 device using Rapid Run v2 sequencing chemistry and flow cells as recommended by the manufacturer (Illumina Inc., San Diego, CA, USA). Paired end 50 bp sequencing runs were completed and the data was converted to the FASTQ Sanger format. Trim Galore was used to remove residual adapter sequences from the reads (https://www.bioinformatics.babraham.ac.uk). Sequencing reads were aligned to the human reference genome assembly (hg19) using TopHat [66]. Further analysis was performed according to workflow for Cufflinks version 2.2.0 and higher [67]. Transcript assembly and estimation of the relative abundances were carried out with Cufflinks [68] using the following settings: Quartile normalization method for the libraries, blind dispersion method for replicates, and biased correction using canonical hg19 as reference. Cuffmerge and Cuffquant were then performed, followed by final comparison analysis in Cuffdiff. Transcripts that revealed significant and consistent differences between conditions in our dataset (*p*-value < 0.05) were subjected to further interpretation by ingenuity pathway analysis. Differentially expressed genes (DGE) were grouped in functional pathways using ingenuity pathway analysis (IPA Spring Release 2017, QIAGEN, Hilde, Germany) on a gene level, using RefSeq annotation [26,56].

### 4.18. Animals

Experiments were performed on 8–12 week-old female BALB/c (24 mice in total) and C57BL/6 mice (36 mice in total). All mice originated from the Centre for Experimental Medicine of the Medical University of Białystok, Poland, and were kept in the animal facility at the Nencki Institute of Experimental Biology throughout the course of the study. After delivery to the Nencki Institute animal facility, mice rested for at least two weeks for acclimatization before starting the experiment. Experiments were approved by the First Local Ethics Committee in Warsaw (permit No. 491/2013 and 542/2018). Animal experimentation was performed in accordance with EU directive 2010/63/EU, and all experiments were undertaken in such a way as to minimize discomfort and the number of animals used in the study.

### 4.19. Ear Pinna Injury and IM Peptide Treatment

Mice were placed individually in separate cages, weighed, and randomly divided into two groups within each mouse strain: The treatment group and the control group. Before the study all mice were randomly given numerical equivalents, and all experimenters performing further analysis were not aware of the experimental group and referred solely to those numbers. Similarly, vials containing IM (treatment) or saline (control) were encrypted and assigned to particular mice, so the person performing injections and ear photographs was not aware of mice background. Lastly, the person quantifying each ear hole closure was not aware of the group. Numerical equivalents of each mice were only decrypted at the end of the study. Before study each animal was anesthetized with Isoflurane (Iso-Vet; Piramal, UK), ears were quickly disinfected with 70% isopropanol and circular punches 2 mm in diameter were made in the center of both ear pinnae using disposable biopsy punches (Integra Miltex; Integra Life Sciences, Princeton, NJ, USA). Due to some occasional self-inflicted injuries (tearing off one or both ear lobes) during the course of the study, the measurement data from destroyed lobes were excluded from comparisons of the next time points.

Immediately after pinna lesion, mice in the treatment group received subcutaneous injections of a sterile solution of IM dissolved in saline (0.5 mg/mL), at a dose of 5.0 µg/g body weight, whereas mice in control groups received equivalent volumes of sterile saline. After recovery, all mice were returned to their home cages. Subcutaneous injections of IM solution or saline, respectively, were continued in the mice on days 1, 2, 3, 4, 7, 14, 21, 28, and 35 after ear pinna injury. Thus, each mouse received 10 injections in total. All subcutaneous injections of IM or saline were made in nape of the neck.

To monitor the progress of wound healing, ears were photographed on the day of the injury (day 0, before and right after pinna injury), and on days 7, 14, 21, 28, 35, and 42. To this end, the mice were anesthetized, their ears were gently flattened using glass cover slips on the flat surface of a sterile plastic block and each ear was photographed from the dorsal side parallel to the glass. A millimeter scale was present within each photographed area for length reference and to allow standardized comparison between each measuring point and between mice. Finally, the area of the wound hole was outlined by hand on digitized images as an overlay, and the hole areas in the ears were evaluated using computer-assisted image analysis (ImageJ Software [69]). Experiments were conducted separately on BALB/c and C57BL/6 mice strain in an identical manner. In total, 12 mice (24 ears) per treatment group and 12 mice (24 ears) per control group entered the analysis in case of BALB/c strain. The final number of analyzed ears was 23 per group on day 42. In C57BL/6 strain, the total number of mice entering the analysis was 8 in the treatment group and 18 in the control group. The final number of analyzed ears on day 42 was 32 in the control group and 16 in the treatment group.

### 4.20. Tissue Isolation for Histological Analyses

On day 42 post-injury, the mice were anesthetized with isoflurane and euthanized by cervical dislocation. The ear pinna tissues were dissected, transferred to Petri dishes, a small microscopic cover glass was placed with small spacers above the pinna to prevent further tissue rolling, and all tissue samples were fixed overnight in 4% paraformaldehyde solution buffered with 0.1 mM PBS in preparation for histopathological analyses. Next, the skin tissue was embedded in paraffin, fixed and cut into 10 µm-thick sections. The morphology of the skin slices was investigated by standard hematoxylin and eosin staining, followed by microscopic analysis using an image analysis system (Olympus BX61VC, Tokyo, Japan).

### 4.21. Statistics

Statistical significance was determined with a non-parametric Mann–Whitney U test. Statistical significance was accepted to be p≤ 0.05. Analyses were performed with Statistica 13.3 software (Statsoft, Warsaw, Poland) and graphs were prepared with GraphPad Prism 8 (GraphPad Software, La Jolla, CA, USA).

## 5. Conclusions

The study presents a complex evaluation of the peptide RDKVYR (IM) as a potential wound healing agent. We demonstrated that IM stimulated growth of human skin cells, showed high immunological safety, low cytotoxicity, and significant pro-proliferative properties. The experiments we performed in mice, using the ear punch wound model, showed that IM moderately promoted tissue repair. The molecular mechanism of IM remains unknown, however its conformational flexibility—revealed in our investigations—and a number of transcriptional responses we observed indicate that the peptide is likely to act on multiple receptors.

## 6. Patents

The results described in this work were included in the Polish patent application number: P.425351

## Figures and Tables

**Table 1:** Functions predicted to be altered in ASCs from donor 6 stimulated with 0.1 µg/mL IM peptide, in comparison to cells cultured with FBS-deprived medium. The results were obtained with disease and function mode implemented in ingenuity pathway analysis (IPA) software.

Function	^1^*p*- value	Predicted Direction of Change	z-Score	Number of Altered Transcripts
Activation of cells	3.84 × 10^−3^	Activation	2.685	21
Immune response	1.33 × 10^−4^	Activation	2.628	25
Chemotaxis	3.57 × 10^−7^	Activation	2.555	29
Cell movements	1.48 × 10^−6^	Activation	2.455	68
Synthesis of nitric oxide	6.62 × 10^−4^	Activation	2.391	9
RNA transactivation	1.22 × 10^−3^	Activation	2.318	20
Cell migration of granulocytes	1.67 × 10^−3^	Activation	2.316	12
Permeability of endothelium cells	2.15 × 10^−3^	Activation	2.193	5
Binding of neutrophils	2.82 × 10^−3^	Activation	2.168	6
Migration of endothelium cells	1.21 × 10^−5^	Activation	2.149	22
Secretion of lipids	3.45 × 10^−3^	Activation	2.122	7
Cell migration of neutrophils	4.48 × 10^−4^	Activation	2.099	11
Migration of cells	1.78 × 10^−4^	Activation	2.063	55
Synthesis of fatty acids	1.12 × 10^−3^	Activation	2.021	13

**^1^**. Algorithm in IPA designed to reduce the chance that random data will produce a significant prediction. It identifies functions with the strongest prediction for increase (positive z-score) or decrease (negative z-score). Values of *p*- value < 0.05 and z-score ≤ −2 or ≥ 2 are considered significant.

## Supplementary Materials

The following are available online. Figure S1. IM peptide stability studies in water and human plasma during 24 h of incubation; Figure S2. Chromatogram comparison of IM peptide before incubation, mixture of peptide and plasma right after mixing, peptide incubated in human plasma for 1 h and human plasma itself; Figure S3. Mass spectra of analyzed fractions of the IM peptide–supernatant (A), last wash (B) and elution fraction (C); Figure S4. The fingerprint region of TOCSY spectrum (80 ms) for IM peptide in water solution at 30 °C; Figure S5. IM cytotoxicity towards 46BR.1N fibroblasts, HaCaT keratinocytes and ASCs analyzed with LDH cytotoxicity assay which measures LDH activity in culture supernatant; Figure S6. Influence of IM peptide on neural cells viability; Figure S7. Effect of IM on cell migration (A), chemotaxis (B) and representative pictures of scratch area (C) in migration test performed with ibidi culture inserts after stimulation with IM peptide for 24 h; Table S1. Protein and cytokine levels in culture supernatants after stimulation with IM; Figure S8a. Transcriptional responses to IM in primary fibroblast cell cultures for a panel of genes controlling cell proliferation potential; Figure S8b. Transcriptional responses to IM in primary keratinocyte cell cultures for a panel of genes controlling cell proliferation potential; Figure S8c. Transcriptional responses to IM in adipocyte stem cell (ASC) cultures for a panel of genes controlling cell proliferation potential; Figure S9. Transcriptional responses to IM in primary fibroblast cell cultures for a panel of genes regulating DNA methylation (DNMT1, DNTMT3A, DNMT3B) and demethylation (TET1, TET2, TET3, TDG); Figure S10. IPA regulatory network (a), with the highest consistency score, in ASC cells isolated from donor 6 and stimulated in vitro with IM, (b) in ASC cells obtained from donor 11 and stimulated with IM and (c) in ASC cells harvested from donor 12 and stimulated with IM; Figure S11. MTT proliferation tests for T47D breast cancer cells treated with IM peptide for 48 or 72 h.; Figure S12. MTT proliferation tests for MCF-7 breast cancer cells treated with IM peptide for 24, 48 or 72 h; Figure S13. HPLC and MS data indicating purity and identification of IM peptide; Figure S14. Confirmation of ASCs “stemness” - flow cytometric analysis of key surface positive (A) and negative (B) markers; Table S2. Proton chemical shifts (ppm) of IM peptide in 90% H2O and 10% D2O at 30 °C; Table S3. Information about donors of cells used for transcript levels analysis; Table S4. PCR primer; Table S5. Information about donors of cells used for transcriptome analysis.
