# Supplementary material for: Imunofan—RDKVYR Peptide—Stimulates Skin Cell Proliferation and Promotes Tissue Repair
Source: Molecules. 2020 Jun 23;25(12):2884. doi: 10.3390/molecules25122884 (PMC7355430; doi:10.3390/molecules25122884)
Supplement: Supplementary file 1 [file molecules-25-02884-s001.zip › supplementary materials/molecules-825828-supplementary.docx]

Imunofan − RDKVYR peptide − stimulates skin cell proliferation and promotes tissue repair

Justyna Sawicka^1+^, Maria Dzierżyńska^1+^, Anna Wardowska^2^ , Milena Deptuła^2^, Piotr Rogujski^3,4^, Paweł Sosnowski^5^, Natalia Filipowicz^6,7^, Alina Mieczkowska^6^, Piotr Sass^5^, Anna Pawlik^8^, Aleksandra Hać^8^, Adriana Schumacher^9^, Magdalena Gucwa^6^, Natalia Karska^1^, Jolanta Kamińska ^5^, Rafał Płatek^3^, Jarosław Mazuryk^3,10^, Jacek Zieliński^11^, Karolina Kondej^12^ Piotr Młynarz^13^, Piotr Mucha^14^, Piotr Skowron^15^, Łukasz Janus^16^, Anna Herman-Antosiewicz^8^, Paweł Sachadyn^5^, Artur Czupryn^3^, Arkadiusz Piotrowski^6,7^, Michał Pikuła^2^*, Sylwia Rodziewicz-Motowidło^1^*

**+**These authors contributed equally to this work

* These authors jointly supervised this work, and are corresponding authors

^1^ Department of Biomedical Chemistry, Faculty of Chemistry, University of Gdańsk, 80-308 Gdańsk, Poland; justyna.sawicka@ug.edu.pl (J.S.); maria.dzierzynska@ug.edu.pl (M.Dz.); natalia.karska@ug.edu.pl; s.rodziewicz-motowidlo@ug.edu.pl (S.R-M.)

^2^ Laboratory of Tissue Engineering and Regenerative Medicine, Department of Embryology, Medical University of Gdańsk, 80-210 Gdańsk, Poland; anna.wardowska@gumed.edu.pl (A.W.); milenadeptula@gumed.edu.pl (M.D.); pikula@gumed.edu.pl (M.P.)

^3^ Laboratory of Neurobiology, Nencki Institute of Experimental Biology, Polish Academy of Sciences, 02-093 Warsaw, Poland; artur@nencki.gov.pl (A.C.); r.platek@nencki.gov.pl (R.P.)

^4^ NeuroRepair Department, Mossakowski Medical Research Centre, Polish Academy of Sciences, 02-106 Warsaw, Poland; progujski@imdik.pan.pl (P.R.)

^5^ Laboratory for Regenerative Biotechnology, Faculty of Chemistry, Gdańsk University of Technology, 80-233 Gdańsk, Poland; paw.sosno@gmail.com (P.So); piotrsass@gmail.com (Pi.S); psach@pg.edu.pl (P.Sa); jolantakaminska6@gmail.com (J.K)

^6^ Department of Biology and Pharmaceutical Botany, Faculty of Pharmacy, Medical University of Gdańsk, 80-416 Gdańsk, Poland; nata@gumed.edu.pl (N.F.); alina_mieczkowska@gumed.edu.pl (A.M), magdag@gumed.edu.pl (M.G.); arpiotr@gumed.edu.pl (A.P.)

^7^ International Research Agenda 3P-Medicine Laboratory, Medical University of Gdańsk, 80-210 Gdańsk, Poland

^8^ Department of Medical Biology and Genetics, Faculty of Biology, University of Gdańsk, 80-308 Gdańsk, Poland: anna.pawlik@biol.ug.edu.pl (A.Pa.); aleksandra.wiczk@biol.ug.edu.pl (A.Wi.); anna.herman@biol.ug.edu.pl (A.H-A)

^9^ Department of Embryology, Medical University of Gdańsk, 80-211 Gdańsk, Poland.;

Present Address: Department of Pharmacology, Faculty of Medicine Medical University of Gdańsk, Gdańsk, Poland; aschumacher@gumed.edu.pl (A.S.)

^10^ Institute of Physical Chemistry, Polish Academy of Sciences, 01-224 Warsaw, Poland; jmazuryk@ichf.edu.pl (J.M.)

^11^ Department of Surgical Oncology, Medical University of Gdańsk, 80-210 Gdańsk, Poland; jacek.zielinski@gumed.edu.pl (J.Z.)

^12^ Department of Plastic Surgery, Medical University of Gdańsk, 80-210 Gdańsk, Poland; kondej@gumed.edu.pl (K.K.)

^13^ Department of Bioorganic Chemistry, Wrocław University of Technology, 50-370 Wrocław, Poland; piotr.mlynarz@pwr.edu.pl (P.Mł.)

^14^ Department of Molecular Biochemistry, Faculty of Chemistry, University of Gdańsk, 80-308 Gdańsk, Poland; piotr.mucha@ug.edu.pl (P.M.)

^15^ Department of Molecular Biotechnology, Faculty of Chemistry, University of Gdańsk, 80-308 Gdańsk, Poland; piotr.skowron@ug.edu.pl (P.Sk.)

^16^ MedVentures Company, 60-141 Poznań, Poland; j.medventures@gmail.com (Ł.J.)

**SUPPLEMENTARY MATERIALS**

**Materials and Methods**

***Confirmation of ASCs “stemness”***

In order to confirm ASCs “stemness” cells form each donor during the 2^nd^ passage were subjected to flow cytometric analysis of key surface markers and analysis of adiopogenic, osteogenic and chondrogenic potential.

***Flow cytometric analysis of surface markers.*** After the 2nd passage the analysis of key surface markers CD31, CD34, CD45, CD13, CD29, CD44, CD49, CD73, CD90, CD105 was performed with flow cytometry. Cells were trypsinised, washed with PBS and stained with monoclonal antibodies for 30 min at RT. Then, they were washed with PBS and analyzed with the BD LSRFortessa flow cytometer.

***Adipogenic and Osteogenic Differentiation****.* For adipogenic and osteogenic differentiation, ASCs were cultured for two weeks in 96-well plates (BD, cat.no. 353872) containing 5 × 10^3^ cells per well in differentiating media (StemPro Adipogenesis Differentiation kit A100070-01, StemPro Osteogenesis Differentiation kit A100072-01, Gibco by Life Technology). Cells cultured in MesenPRO RS medium (cat. 12746012, Gibco by Life Technology) were used as control. The medium was changed twice per week. At the end of a differential culture, the medium was removed, the cells were washed in PBS and fixed with 3,7% formaldehyde. To confirm adipocyte (cells with red–stained lipid vesicles) and osteocyte (red-staining calcium deposits in the medium) differentiation the cells were stained with Oil Red O (Sigma, cat. O0625) and Alizarin Red S (Sigma, cat. A5533) respectively according to manufacturer’s instructions. The cells were observed using a Phase-contrast fluorescent microscope (Zeiss, Oberkochen, Germany) and photographed (Zeiss, AxioVision Software, Oberkochen, Germany).

***Chondrogenic Differentiation***. Chondrogenesis differentiation was checked by micropellet formation. 5 × 10^5^ cells were placed in a 15 ml conical tube and centrifuged at 1500 rpm for 5 min. The pellet was cultured in differentiating medium (StemProChondrogenesis Differentiation kit, Gibco by Life Technology) for six weeks. During the culturemedium was changed twice a week. The pellet was fixed with 3,7% formaldehyde for 24 h at room temperature (RT), dehydrated by in increasing ethanol concentrations and embedded in paraffin. Histological slides have been prepared and stained with 1% Alcian Blue pH 2.5 (Sigma-Aldrich, Saint Louis, Missouri, USA) in 3% acetic acid for 30 min at RT (blue staining indicating synthesis of proteoglycans by chondrocytes) and counterstained with Harris’s hematoxylin (Sigma-Aldrich, Saint Louis, Missouri, USA) for 1 min at RT.

***Cancer cell prolifertion assay.*** Cell proliferation was determined by the MTT method. MTT test measures enzymatic activity, which is a marker of viable cells. Drop in amount of formazan informs that there is less metabolically active cells as compared with control (taken as 100%); however, it might be caused by cell cycle arrest (stopped or slowed proliferation) and/or cell death (which also may happen after prolonged incubation with tested compounds). T47D or MCF-7 breast cancer cells were cultured in RPMI-1640 supplemented with 10 % fetal bovine serum and 1 % penicillin/streptomycin solution. Cells were seeded, at a density of 2 x 10^3^ per well, in a 96-well plate and allowed to attach overnight. The medium was replaced with fresh medium supplemented with desired concentrations of each investigated compound for 24, 48 or 72 h. Before the end of treatment, 25 µL of MTT solution (4 mg per ml^‑1^) were added to each well. After 3 h of incubation, medium was removed, and formazan crystals were dissolved in 100 µL of DMSO. Absorbance was measured at 570 nm (with reference wavelength 660 nm) in a Victor microplate reader. Data were obtained from at least three independent experiments performed in triplicate.

***Neural viability assessments.*** Primary cortical neural cultures were obtained from E18.5 Wistar rat embryos. For this purpose, the rat was euthanized in a CO_2_ chamber. The cortex tissue was immediately excised from the brain, moved to a HBSS medium (diluted 10 × and supplemented with a penicilin/streptomycin mixture), and washed precisely. Afterwards, the tissue was trypsinized for 10 min at 37 ºC, and homogenized gently. The cells were centrifuged in 300 rcf for 4 min and suspended in a Neurobasal medium (supplemented with 1 mM sodium pyruvate, final glucose concentration of 12.5 mM), supplemented with 2 % B27, 200 mM L-glutamine, 10 mM glutamic acid, a penicilin/streptomycin mixture), and seeded in a 96-well and 24-well plate (both PDL-coated, Corning) in a number 15.000 and 200.000 cells per well, respectively. The neural cultures were incubated in a CO2 chamber (5 %, 95 % air balance, 98 % humidity, 37 ºC) for 14 days and were used on day in vitro 14-15. Neural viability was quantitatively examined by CellTiterBlue® (CTB) assay (Promega, G8080) after 20-hour long incubation of the cells at 37 ºC in the CO2. The test was performed according to the manufacture guideline, which involved adding of the agent solution to the neural culture wells followed with the incubation for 1-4 h at 37 ºC (5% CO_2_). The viability test itself was optimized in 96-well PDL-coated plates (BioCoat, Corning) prior to all assessments. The assays basis relies on the conversion of resazurin (dark blue color) to resorufin (pink color) in mitochondria of living cells. Fluorescence intensity of the CTB reagent is measured spectrofluorymetrically (560_ex_/590_em_) and is proportional to the number of viable cells. No insult sample referred as a 100% viability control and the data from the insulted cells was quantified and presented relatively as mean ± SD. The data was analyzed by a Mann-Whitney test with p-value < 0.05 values considered statistically significant. Four to six wells were used in all assays with sister neural cultures and repeated a minimum of three times independently.

***Influence of Imunofan on neural viability.*** Influence of Imunofan on neural viability was assessed by exposing the cells to different peptide concentrations (1, 10, 100, 200 µM) for 20 h at 37 ºC in a 5% CO2 chamber. The assessments were performed in 24-well PDL-coated plates (BioCoat, Corning) with working volume of 500 µl/well. The respective peptide solutions were prepared in culture medium so that only 50 µl of the medium was exchanged in all wells, including control, untreated samples. Four wells were exploited for each concentration.


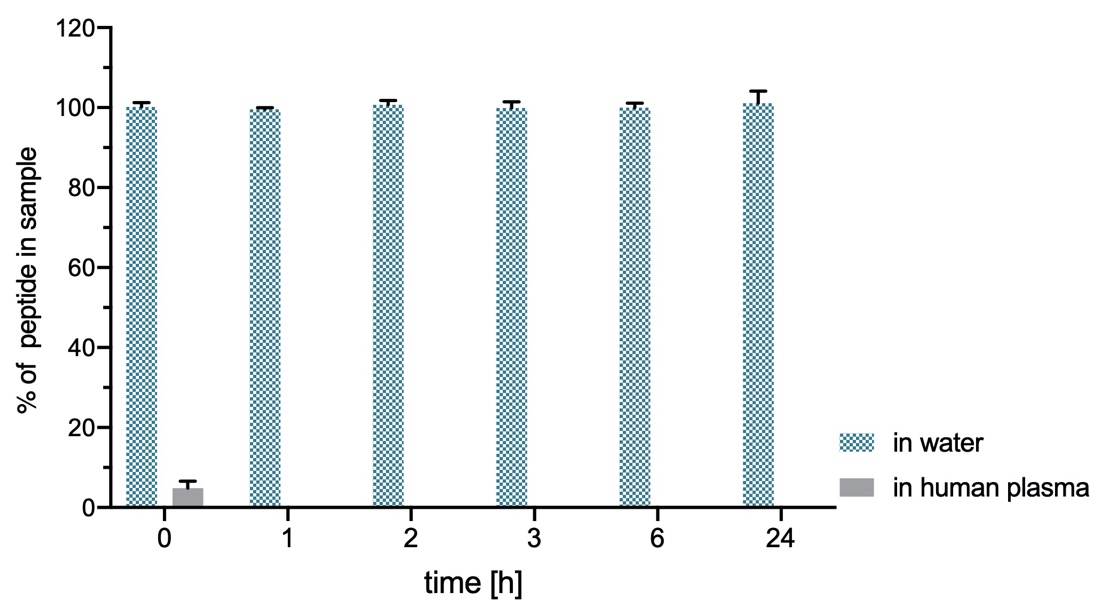


**Figure S1.** IM peptide stability studies in water and human plasma during 24 hours of incubation. The graph shows percentage of peptide remaining in the sample based on HPLC data. The graph shows results from 3 independent experiments (1 replicates in each, n=3). Results are presented as mean with SD.


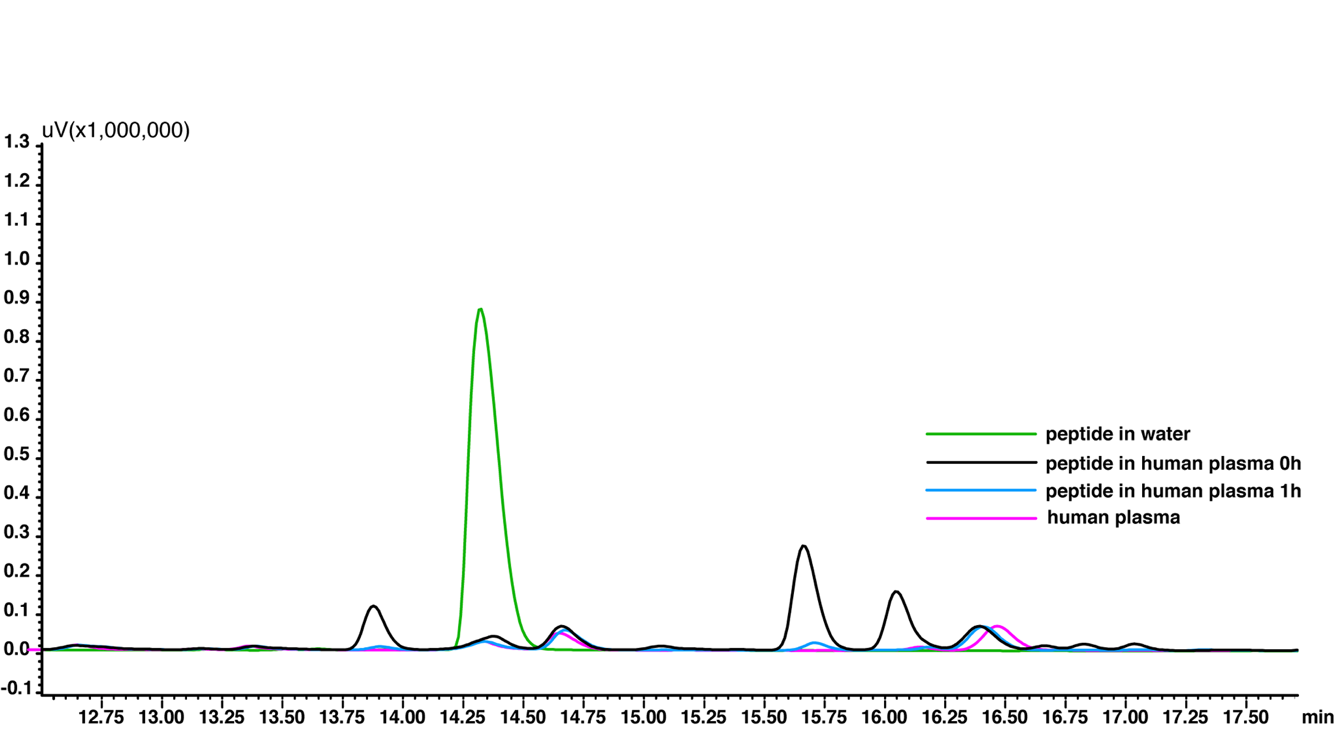


**Figure S2.** Chromatogram comparison of IM peptide before incubation, mixture of peptide and plasma right after mixing, peptide incubated in human plasma for 1h and human plasma itself.


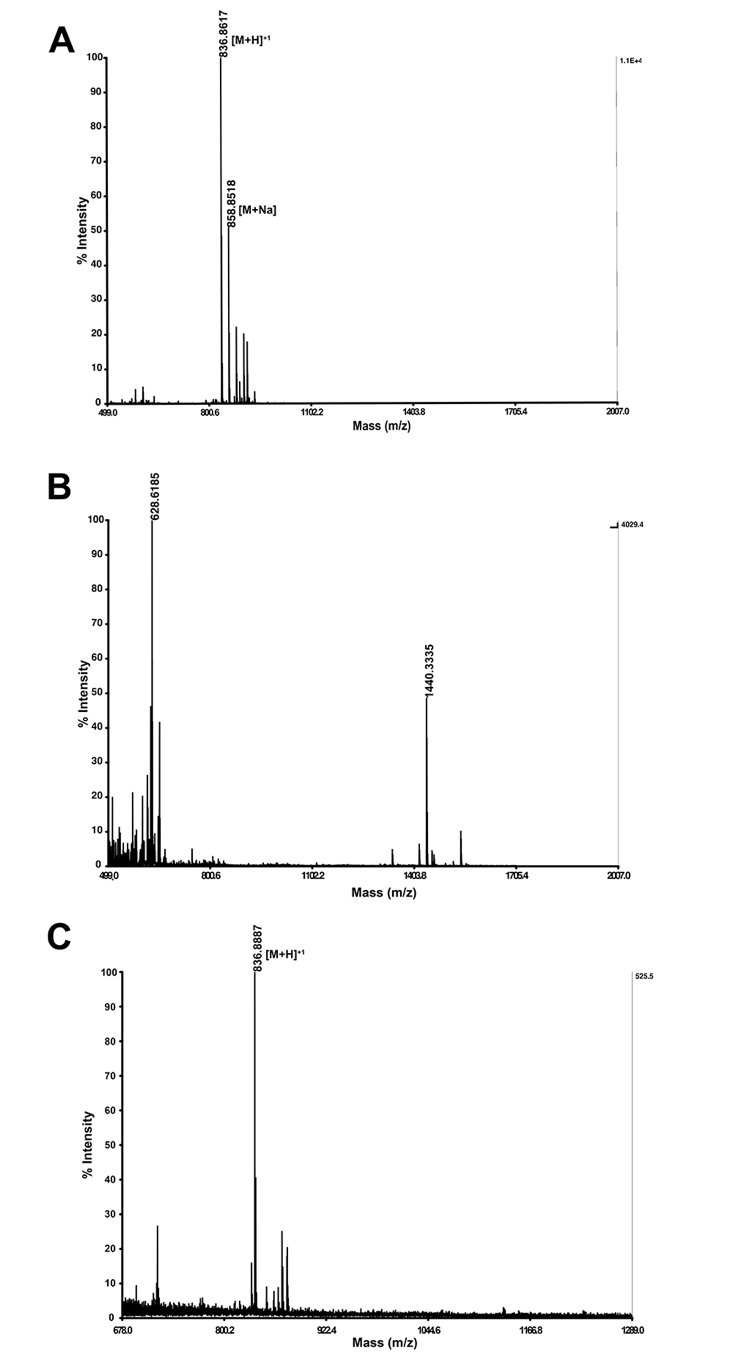


**Figure S3**. Mass spectra of analyzed fractions of the IM peptide – supernatant (A), last wash (B) and elution fraction (C).


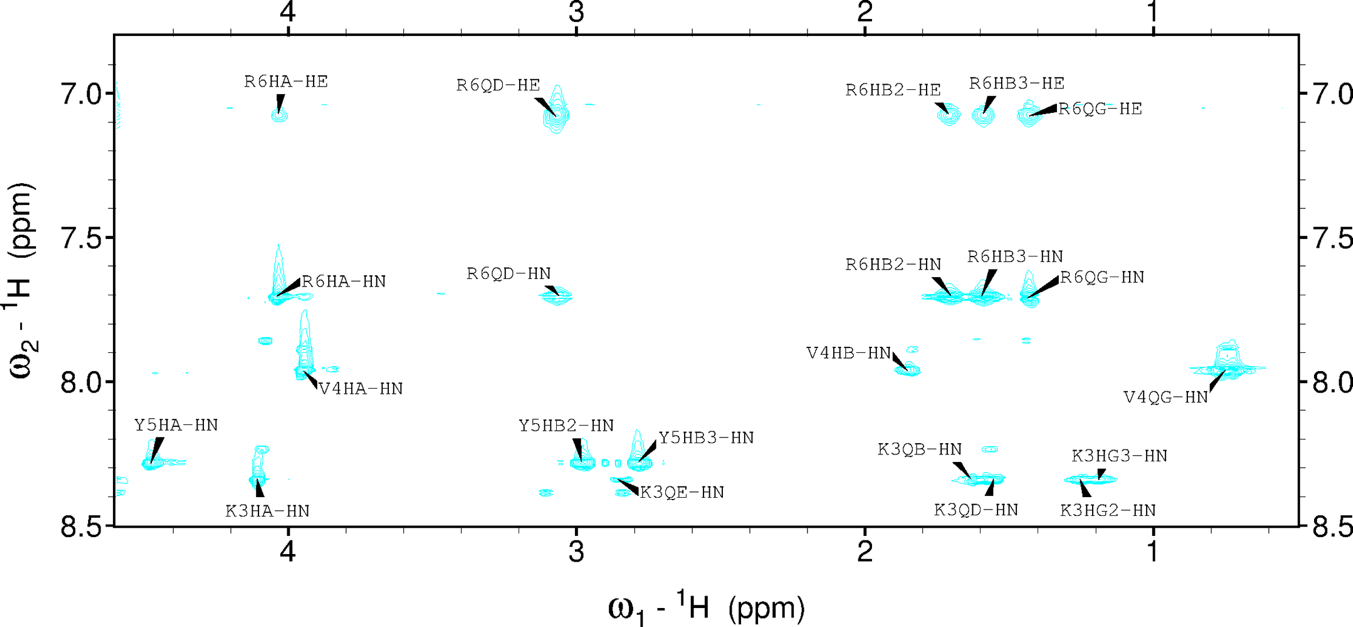


**Figure S4.** The fingerprint region of TOCSY spectrum (80 ms) for IM peptide in water solution at 30°C. Only the major conformation signals on the spectrum were described.


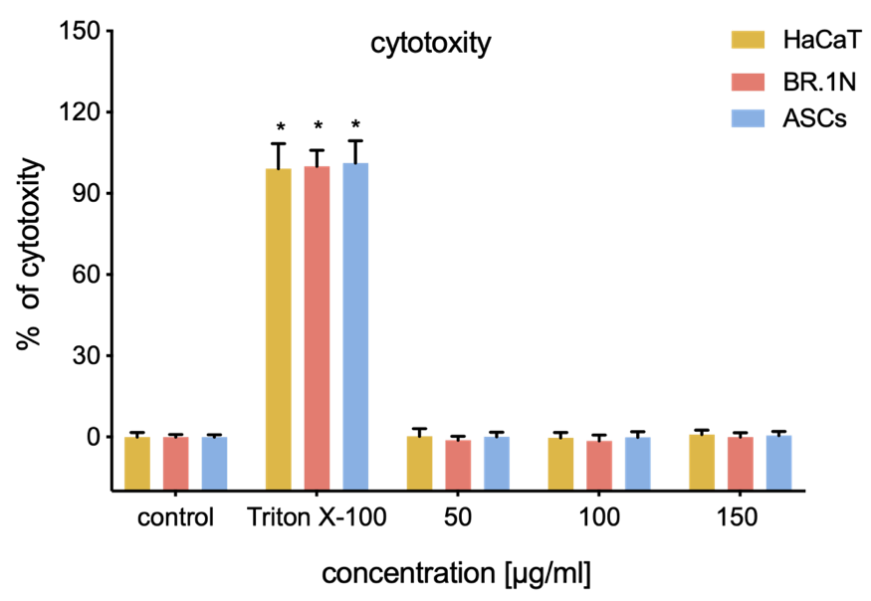


**Figure S5.** IM cytotoxicity towards 46BR.1N fibroblasts, HaCaT keratinocytes and ASCs analyzed with LDH cytotoxicity assay which measures LDH activity in culture supernatant. The graph shows results from 4 independent experiments (4 replicates in each, n=16) for 46BR.1N and HaCaT cells, and results from 3 independent experiments for ASCs (4 replicates in each, n=12). Results are presented as mean with SD. *- statistically significant differences compared to control, Mann-Whitney U test, *p<*0.05. TRITON X- positive control- cells grown in medium containing 1% TRTION X. Maximum LDH release = maximum cytotoxicity.


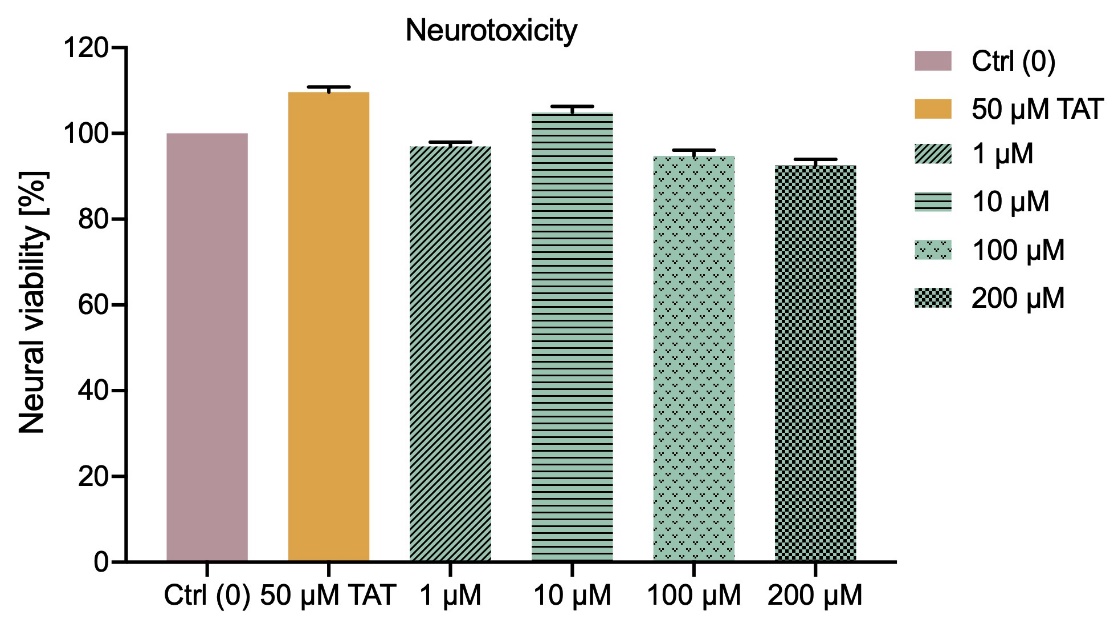


**Figure S6.** Influence of IM peptide on neural cells viability.


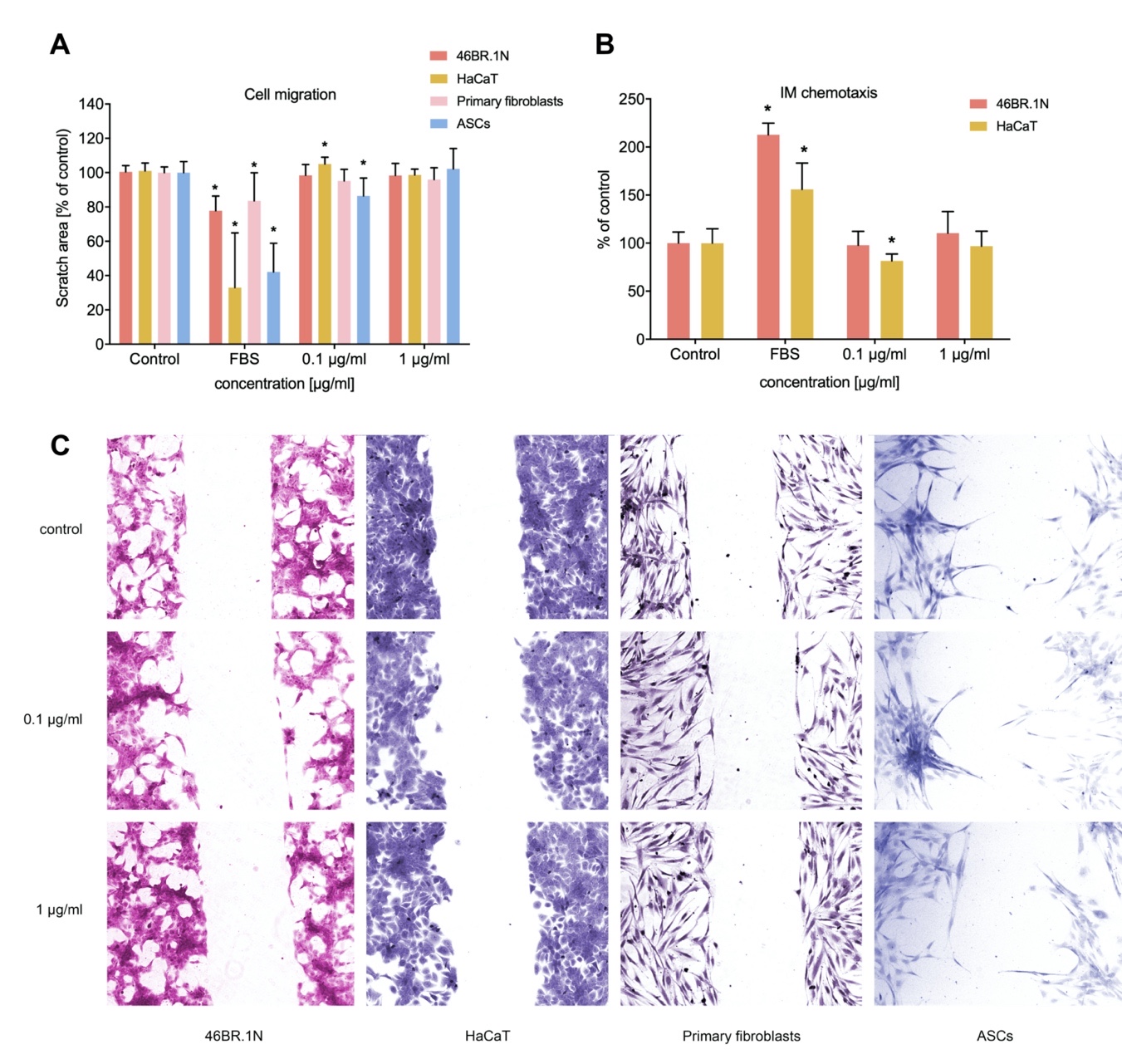


**Figure S7.** Effect of IM on cell migration (A), chemotaxis (B) and representative pictures of scratch area (C) in migration test performed with ibidi culture inserts after stimulation with IM peptide for 24 hours. Graph A shows results from 3 independent experiments for 46BR.1N, HaCaT cells and primary fibroblasts (4 replicates in each, n = 12) and 4 independent experiments for ASCs (4 replicates in each, n = 16). Graph B shows results from 3 independent experiments (4 replicates in each, n = 12). Results are presented as mean with SD. * - statistically significant differences compared to the control, Mann-Whitney U test, *p<*0.05. FBS - positive control - cells grown in medium containing 10% FBS.

**Table S1.** Protein and cytokine levels in culture supernatants after stimulation with IM.

Cytokine/growth factor levels in culture supernatants from primary cells (skin fibroblasts, keratinocytes and ASCs) stimulated with IM peptide (0.1 μg/ml) and analyzed by the Luminex® xMAP® assay containing a mixture of beads specific for each analyte, as described in the “Methods” section. Cytokine/growth factor levels are mean ± SD and expressed in picograms per milliliter. Statistical analyses revealed no significant differences (Mann-Whitney U test, *p<*0.05)

| **Cytokines /**  **growth factors** |  | **Fibroblasts** | **Keratinocytes** | **ASCs** |
| --- | --- | --- | --- | --- |
| **angiopoetin 2** | control | 1.81±3.14 | 1.72±2.04 | 21.70±36.21 |
|  | IM | 2.31±3.00 | 0 | 25.46±49.95 |
| **GCSF** | control | 157,78±148,53 | 8,26±9,34 | 0 |
|  | IM | 153.98±144.52 | 3.75±2.65 | 0 |
| **endoglin** | control | 5.64±9.77 | 0 | 54.61±53.30 |
|  | IM | 4.50±7.80 | 0 | 45.02±73.48 |
| **endothelin-1** | control | 0.09±0.08 | 45.25±16.92 | 0 |
|  | IM | 0.09±0.08 | 30.31±8.72 | 0 |
| **leptin** | control | 77.91±8.89 | 23.61±9.95 | 3.27±6.70 |
|  | IM | 83.04±0.01 | 23.61±9.95 | 7.03±12.93 |
| **follistatin** | control | 1192.77±580.46 | 5212.05±7591.77 | 195.96±35.68 |
|  | IM | 1156.14±585.55 | 3168.96±4575.17 | 269.66±129.36 |
| **IL-8** | control | 1361.04±356.15 | 15.55±4.09 | 98.01±81.44 |
|  | IM | 1185.24±344.35 | 34.02±57.75 | 98.99±82.32 |
| **HGF** | control | 105.19±139.80 | 0 | 276.29±182.83 |
|  | IM | 89.76±110.49 | 0 | 226.25±174.78 |
| **HB-EGF** | control | 0 | 9.05±0.86 | 0 |
|  | IM | 0 | 6.15±2.09 | 0 |
| **PLGF** | control | 39.86±51.08 | 27.09±4.36 | 9.49±1.61 |
|  | IM | 40.54±52.46 | 23.32±1.75 | 9.98±6.68 |
| **VEGF-C** | control | 279.37±59.37 | 122.06±56.99 | 206.15±64.83 |
|  | IM | 259.49±46.53 | 106.28±4.10 | 179.35±122.68 |
| **VEGF-D** | control | 0.72±0.01 | 4.53±5.10 | 0 |
|  | IM | 0.72±0.01 | 1.81±1.54 | 0 |
| **FGF-2** | control | 44.33±26.42 | 3.77±3.27 | 3.83±4.36 |
|  | IM | 39.99±10.47 | 1.89±3.27 | 5.60±7.32 |
| **VEGF-A** | control | 138.09±62.01 | 1823.35±1279.63 | 175.70±63.35 |
|  | IM | 134.89±76.03 | 735.64±410.97 | 196.10±31.81 |
| **NGF** | control | NA | NA | 2.77±0.94 |
|  | IM ***** | NA | NA | 1.38±0.60 |
| **IL-6** | control | NA | NA | 244.24±155.69 |
|  | IM | NA | NA | 205.64±105.10 |
| **MCP-1** | control | NA | NA | 1616.75±1533.98 |
|  | IM | NA | NA | 2147.38±2677.89 |
| **TNF alfa** | control | NA | NA | 0.04±0.07 |
|  | IM | NA | NA | 0.013±0.01 |
| **resistin** | control | NA | NA | 0 |
|  | IM | NA | NA | 0 |
| **PAI-1 (total)** | control | NA | NA | 6017.75±3734.08 |
|  | IM | NA | NA | 5423.75±3038.58 |

mean± SD, NA – not acquired


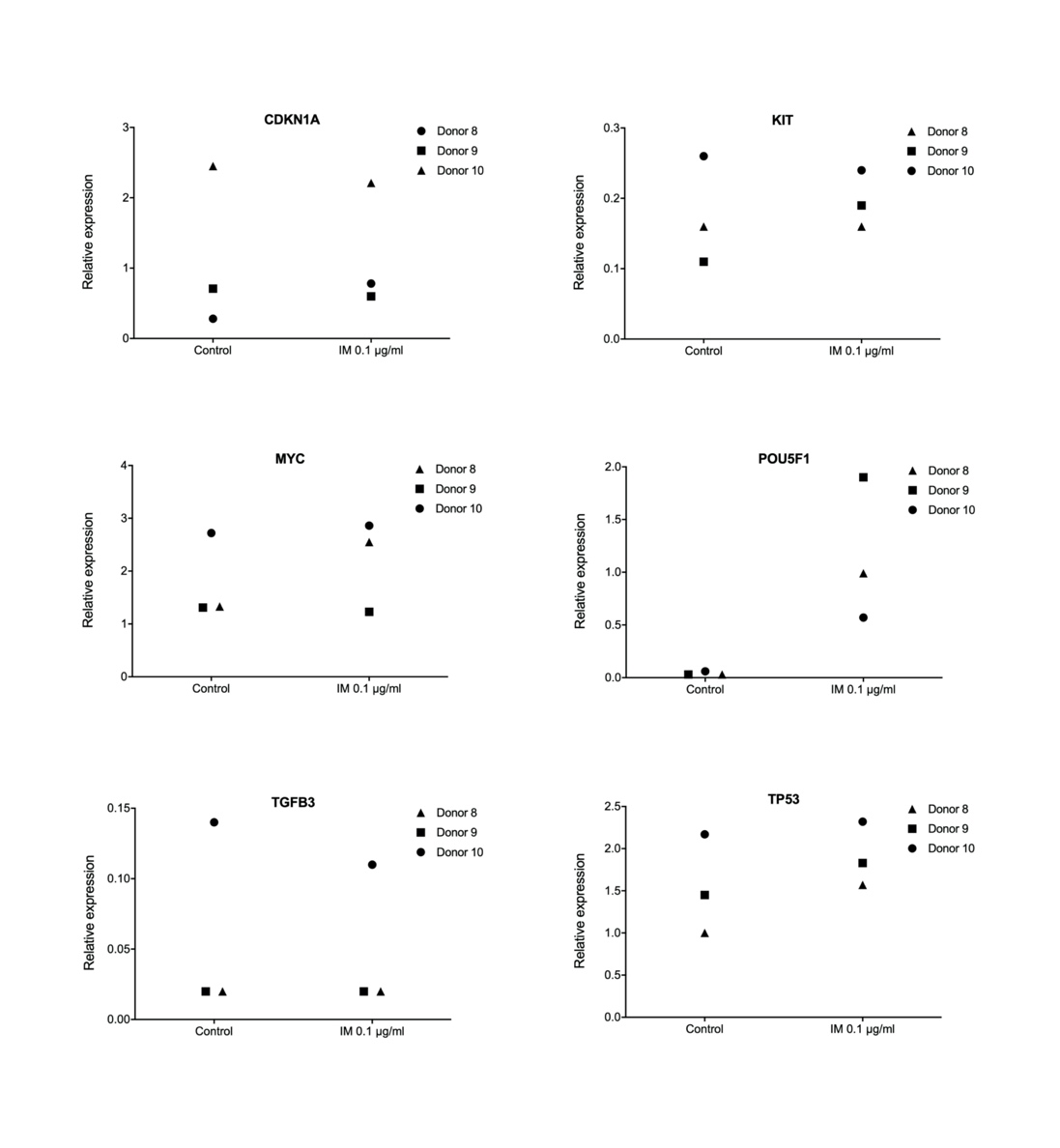


**Figure S8a.** Transcriptional responses to IM in primary fibroblast cell cultures for a panel of genes controlling cell proliferation potential. Controls were cultured in the same medium without IM addition.


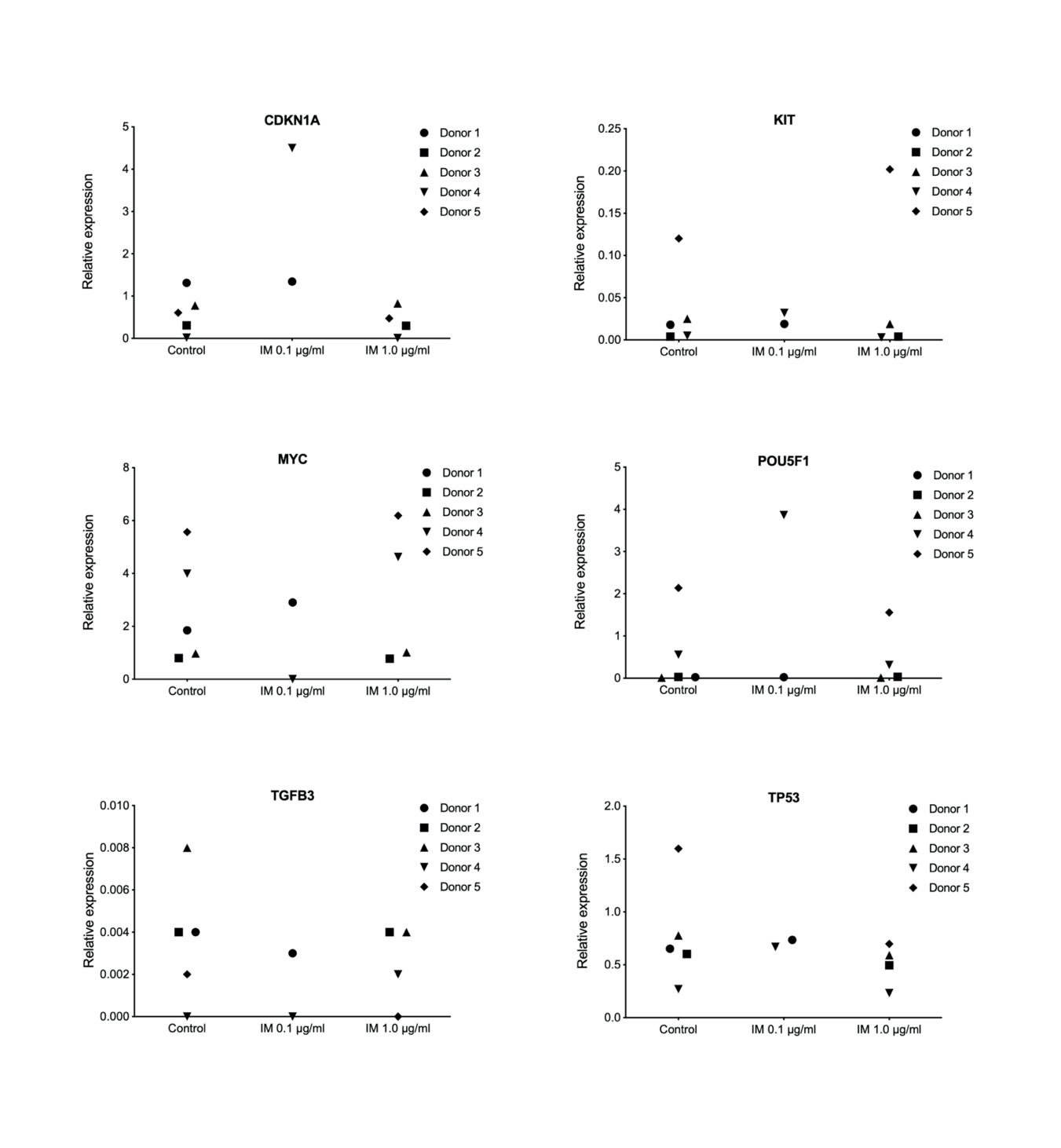


**Figure S8b.** Transcriptional responses to IM in primary keratinocyte cell cultures for a panel of genes controlling cell proliferation potential. Controls were cultured in the same medium without IM addition.


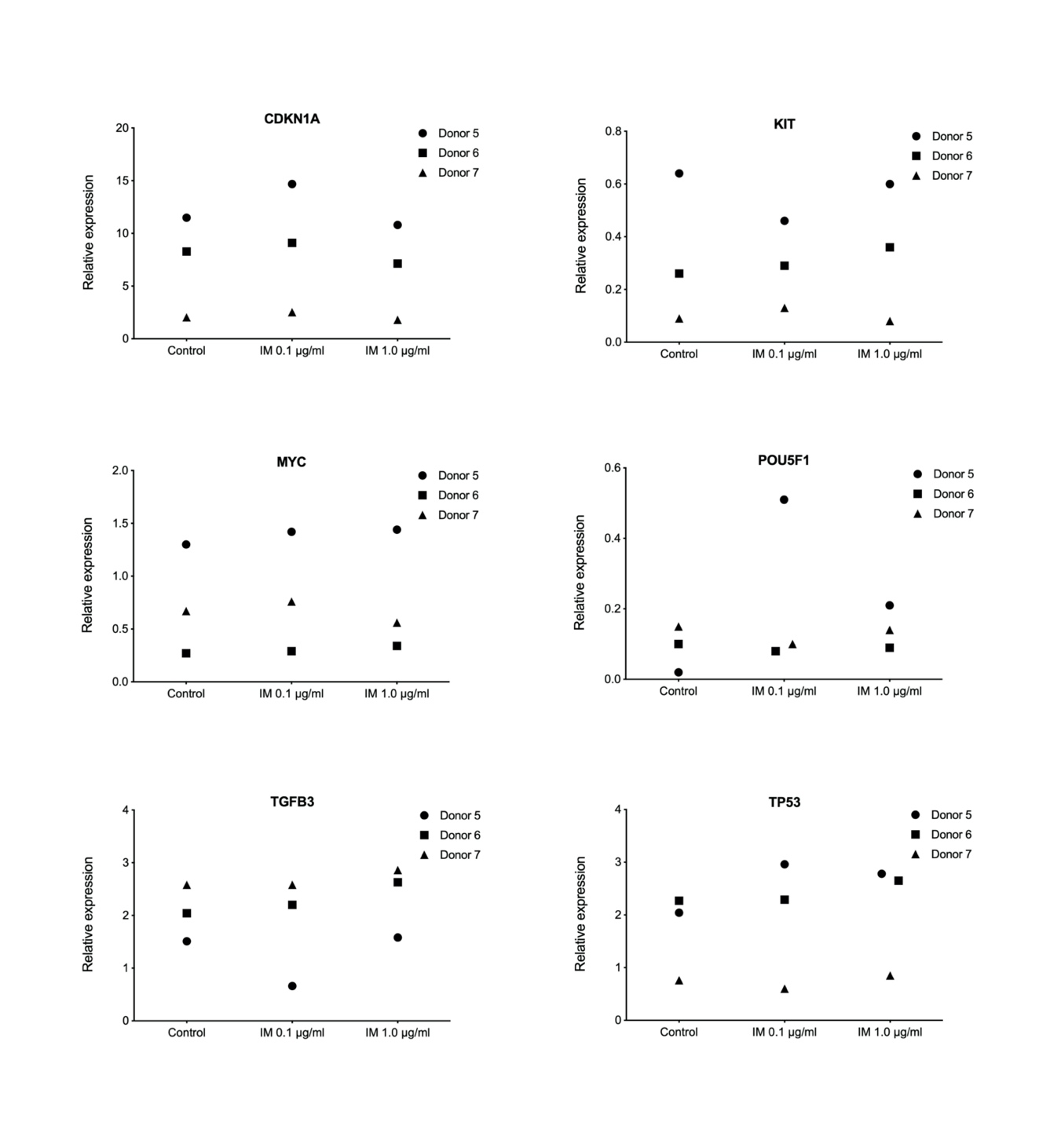


**Figure S8c.** Transcriptional responses to IM in adipocyte stem cell (ASC) cultures for a panel of genes controlling cell proliferation potential. Controls were cultured in the same medium without IM addition.


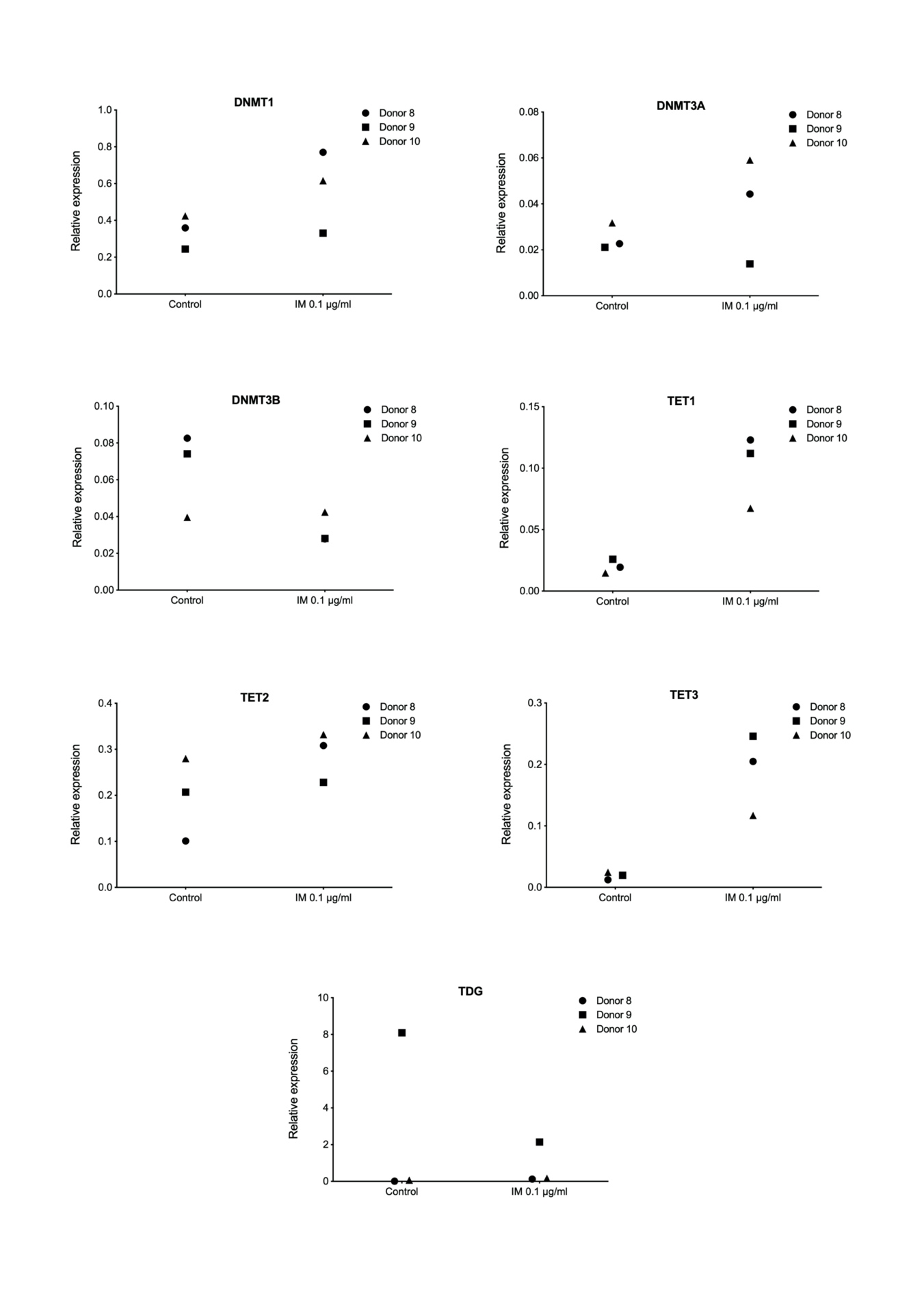


**Figure S9.** Transcriptional responses to IM in primary fibroblast cell cultures for a panel of genes regulating DNA methylation (DNMT1, DNTMT3A, DNMT3B) and demethylation (TET1, TET2, TET3, TDG). Controls were cultured in the same medium without IM addition.


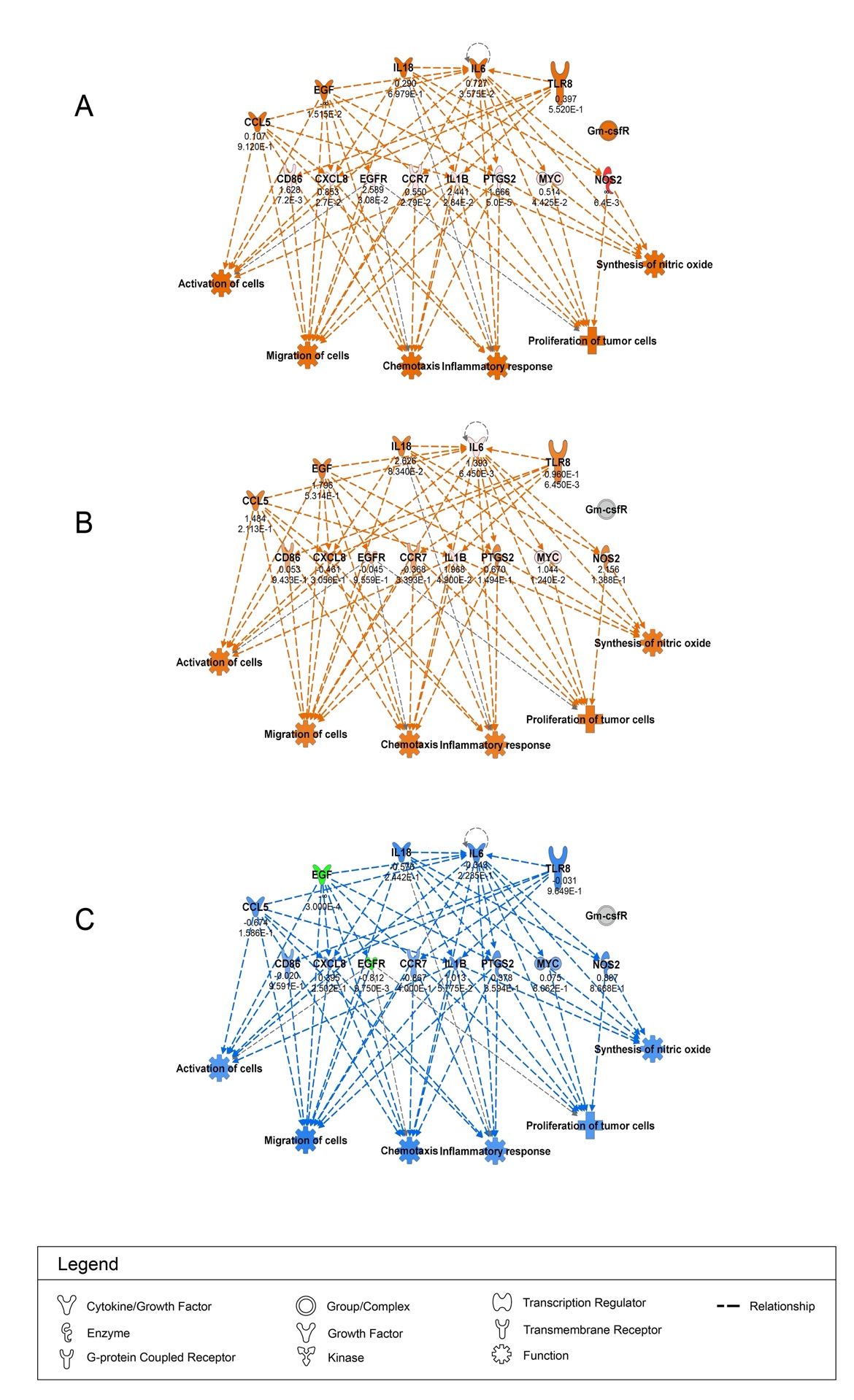


**Figure S10.** IPA regulatory network (a), with the highest consistency score, in ASC cells isolated from donor 6 and stimulated in vitro with IM, (b) in ASC cells obtained from donor 11 and stimulated with IM and (c) in ASC cells harvested from donor 12 and stimulated with IM. The values under the molecule names refer to log2fold change of gene expression calculated in cufflinks package and for p-value, the orange and blue colors of molecules and downstream effects stand for predicted activation or inhibition respectively.


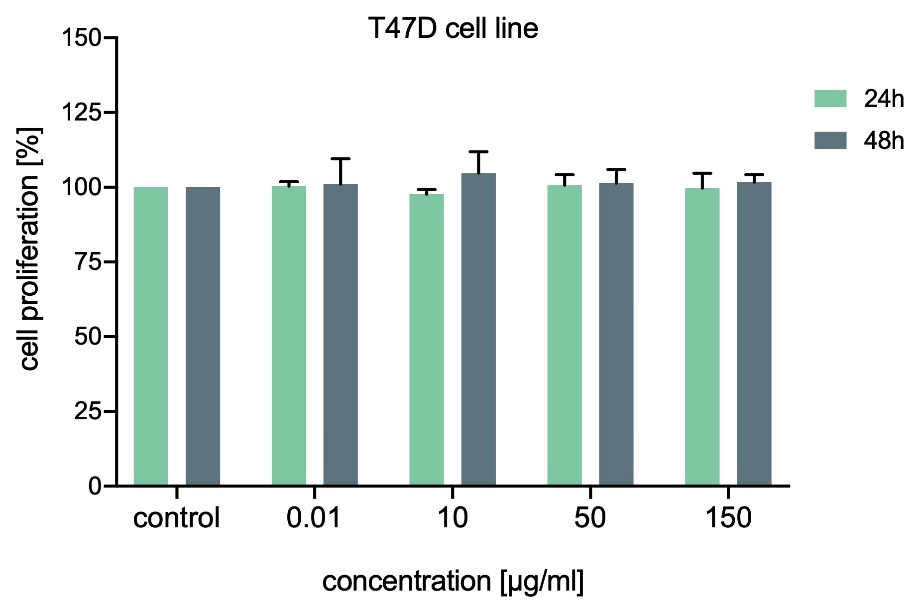


**Figure S11.** MTT proliferation tests for T47D breast cancer cells treated with IM peptide for 48 or 72 h.

Differences are not statistically significant from control (Mann Whitney U-test, p<0,05).


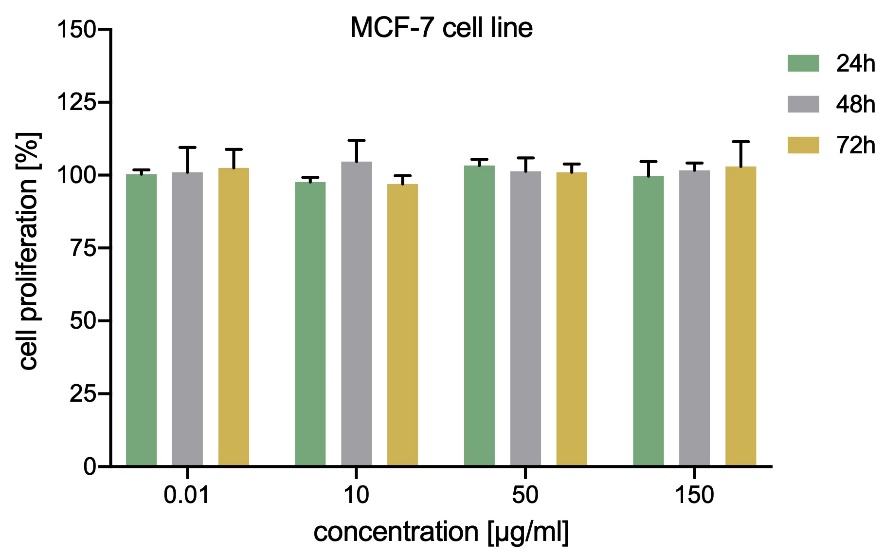


**Figure S12.** MTT proliferation tests for MCF-7 breast cancer cells treated with IM peptide for 24, 48 or 72 h. Differences are not statistically significant from control (Mann Whitney U-test, p<0,05).


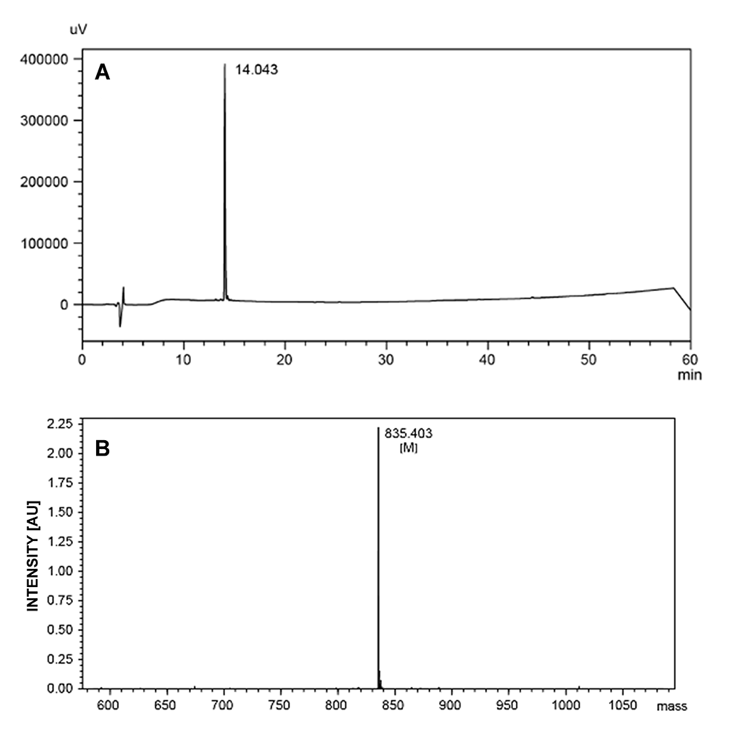


**Figure S13.** HPLC and MS data indicating purity and identification of IM peptide.


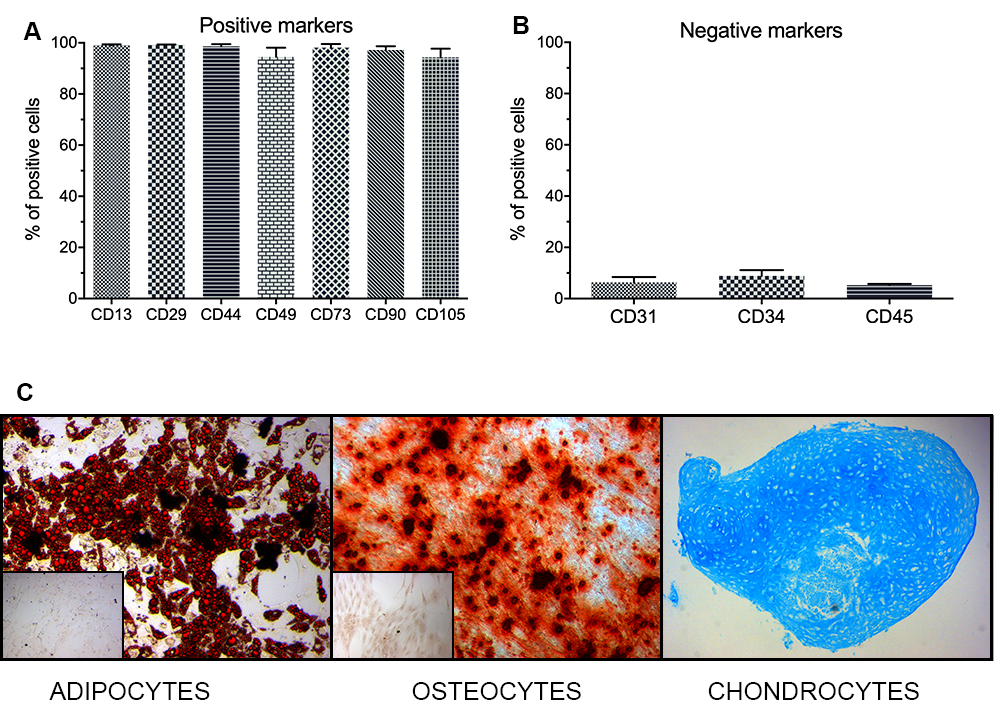


Figure S14. Confirmation of ASCs “stemness” - flow cytometric analysis of key surface positive (A) and negative (B) markers. The graphs represent results of analysis of cells from 4 representative donors, presented as mean±SD of % of positive cells. C- representative images of ASCs differentiation into adipocytes (Oil red staining of lipid droplets), osteocytes (Alizarin red staining of calcium deposits) and chondrocytes (Alcian blue staining of glycosaminoglycans).

**Table S2.** Proton chemical shifts (ppm) of IM peptide in 90% H_2_O and 10% D_2_O at 30°C.

**Proton chemical shifts**

| **Residue** | **HN** | **Hα** | **Hβ** | **Hγ** | **Hδ** | **Others** |
| --- | --- | --- | --- | --- | --- | --- |
| R1 | - | 3.91 | 1.81 | 1.58 | 3.1 |  |
| D2 | - | 4.53 | 2.63  2.49 | - | - | - |
| K3 | 8.34 | 4.11 | 1.61 | 1.26  1.18 | 1.55 | Hε 2.85 |
| V4 | 7.96 | 3.94 |  |  | 1.02 |  |
| Y5 | 8.28 | 4.48 | 2.98  2.65 |  |  | Ar 7.05 |
| R6 | 7.70 | 4.04 | 1.70  1.59 | 1.43 | 3.066 | NHε 7.1 |

**Table S3.** Information about donors of cells used for transcript levels analysis. Abbreviations: surgery clinic: PS- Plastic Surgery, OS- oncological surgery, ex F – female, M – male

| Donor | Surgery clinic | Age | Sex | Material |
| --- | --- | --- | --- | --- |
| Donor 1 | PS | 38 | M | skin |
| Donor 2 | PS | 36 | F | skin |
| Donor 3 | PS | 53 | F | skin |
| Donor 4 | OS | 64 | F | skin |
| Donor 5 | PS | 35 | F | skin, adipose tissue |
| Donor 6 | PS | 47 | F | adipose tissue |
| Donor 7 | OS | 66 | F | adipose tissue |
| Donor 8 | PS | 63 | F | skin |
| Donor 9 | OS | 60 | F | skin |
| Donor 10 | OS | 77 | M | skin |

**Table S4.** PCR primers

| **GENE** | **Forward primer** | **Reverse primer** | **Amplicon size bp** |
| --- | --- | --- | --- |
| *ACTB* | CATGGGTCAGAAGGATTCCT | ACACGCAGCTCATTGTAGAA | 150 |
| *CDKN1A* | CTGGCACCTCACCTGCTCTG | CGGATTAGGGCTTCCTCTTGG | 216 |
| *DNMT1* | GAGCTACCACGCAGACATCA | CGAGGAAGTAGAAGCGGTTG | 161 |
| *DNMT3A* | TATTGATGAGCGCACAAGAGAGC | GGGTGTTCCAGGGTAACATTGAG | 111 |
| *DNMT3B* | CCCATTCGAGTCCTGTCATT | GGTTCCAACAGCAATGGACT | 126 |
| *KIT* | CTGCGTTCTGCTCCTACTGCTT | CCTGGATGGATGGATGGT | 133 |
| *MYC* | ATAGCAGCGGGCGGGCA | CGAGGTCATAGTTCCTGTTGGTG | 245 |
| *POU5F1* | TCAGCCACATCGCCCA | AGACCCAGCAGCCTCAA | 213 |
| *TBP* | TCCACAGTGAATCTTGGTTGTA | CACCATTTTCCCAGAACTGA | 153 |
| *TDG* | AAGATGTGCTCAGTTTCCTCG | TAACAGCCATCTTCTTTGC | 167 |
| *TET1* | CAGAACCTAAACCACCCGTG | TGCTTCGTAGCGCCATTGTAA | 141 |
| *TET2* | GATAGAACCAACCATGTTGAGGG | TGGAGCTTTGTAGCCAGAGGT | 95 |
| *TET3* | TCCAGCAACTCCTAGAACTGAG | AGGCCGCTTGAATACTGACTG | 169 |
| *TGFB3* | CGTGAGTGGCTGTTGAGAAG | GATTAGATGAGGGTTGTGGTGA | 213 |
| *TP53* | GCTTTGAGGTGCGTGTTTGTG | AGTGGTTTCTTCTTTGGCTGGG | 163 |

**Table S5.** Information about donors of cells used for transcriptome analysis. Abbreviations: sex F – female, M – male. ^1^ Only donors with BMI<30 were selected for the analysis to avoid possible obesity-related bias.

| Donor | Age | Sex | BMI^1^ | Diagnosis |
| --- | --- | --- | --- | --- |
| Donor 6 | 47 | F | 26 | Abdomen malformation |
| Donor 11 | 53 | F | 25 | Excess of thigh skin (bariatric patient) |
| Donor 12 | 59 | F | 28 | Breast reconstruction |
