# Supplementary figures and images for: Imunofan—RDKVYR Peptide—Stimulates Skin Cell Proliferation and Promotes Tissue Repair
Source: Molecules. 2020 Jun 23;25(12):2884. doi: 10.3390/molecules25122884 (PMC7355430; doi:10.3390/molecules25122884)

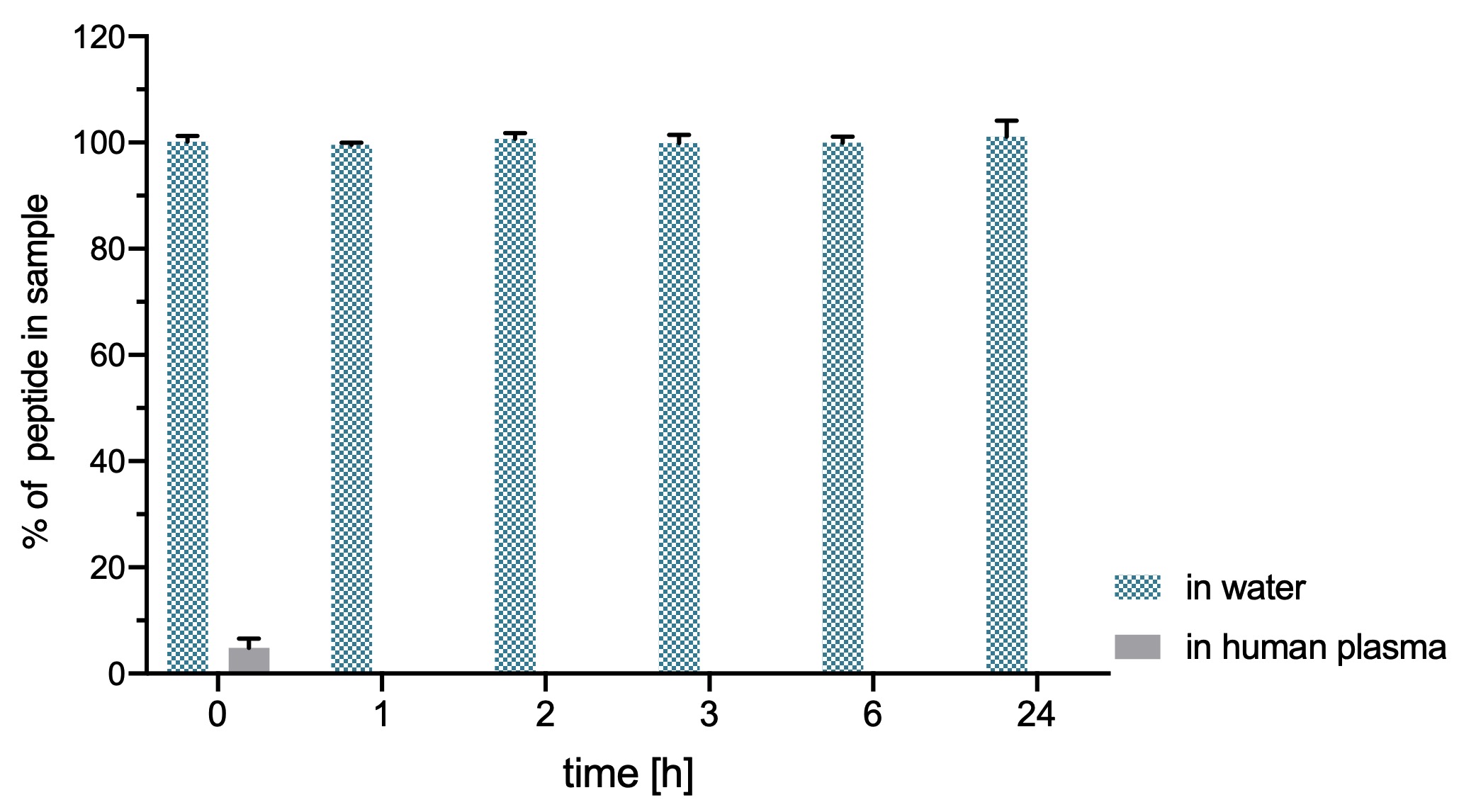

Supplement: Supplementary file 1 [file molecules-25-02884-s001.zip › supplementary materials/Figure S1.jpg]

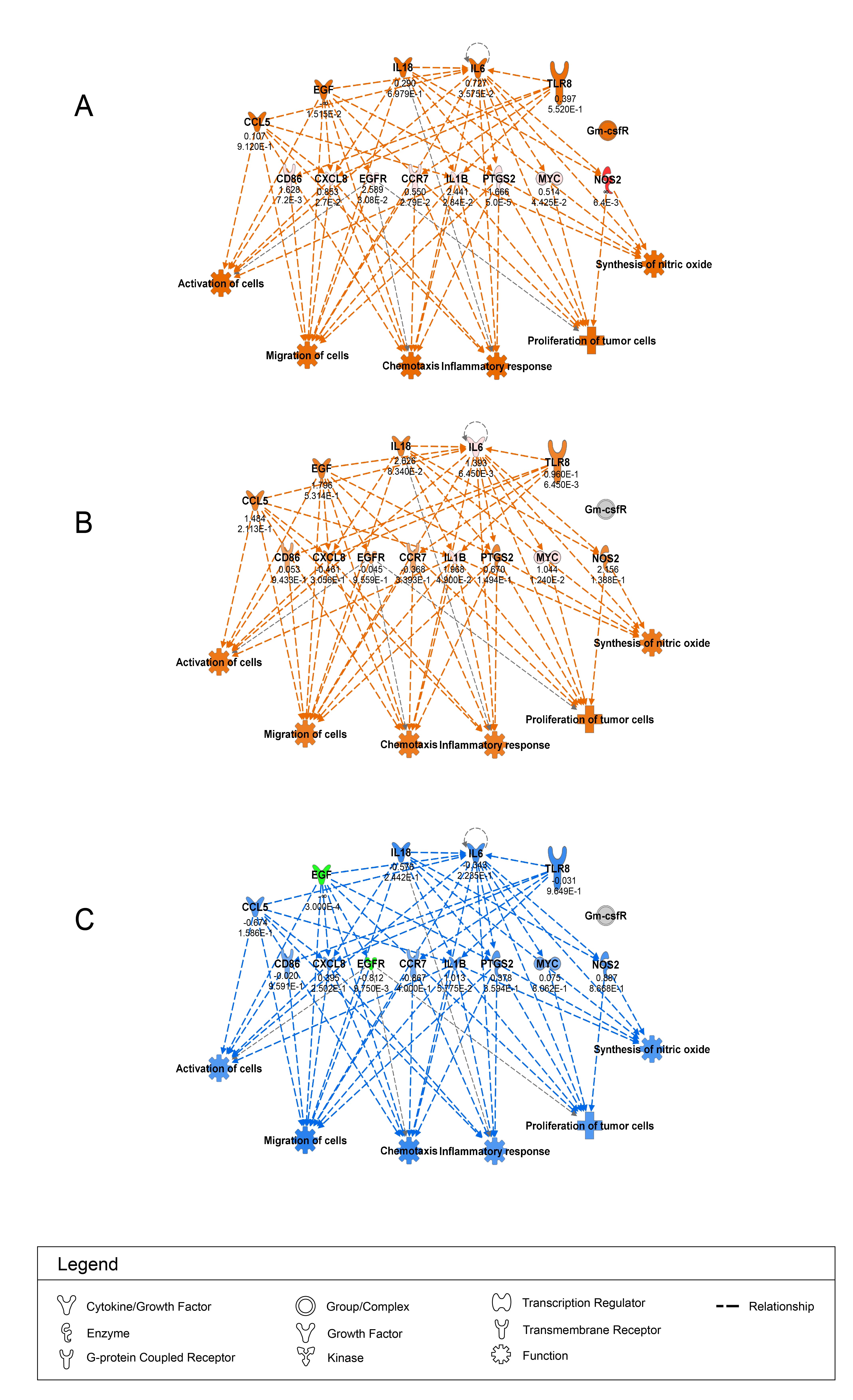

Supplement: Supplementary file 1 [file molecules-25-02884-s001.zip › supplementary materials/Figure S10.jpg]

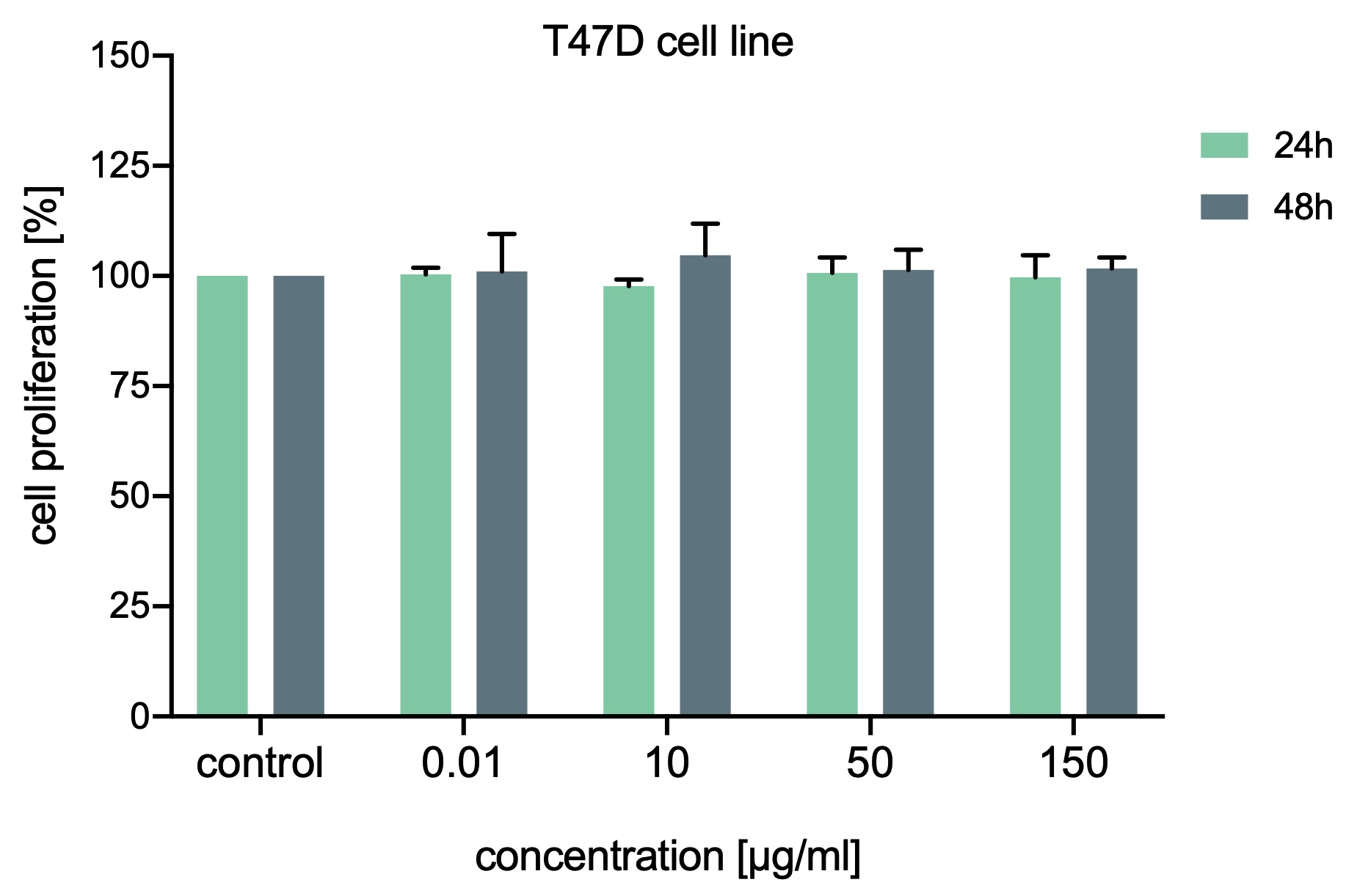

Supplement: Supplementary file 1 [file molecules-25-02884-s001.zip › supplementary materials/Figure S11.jpg]

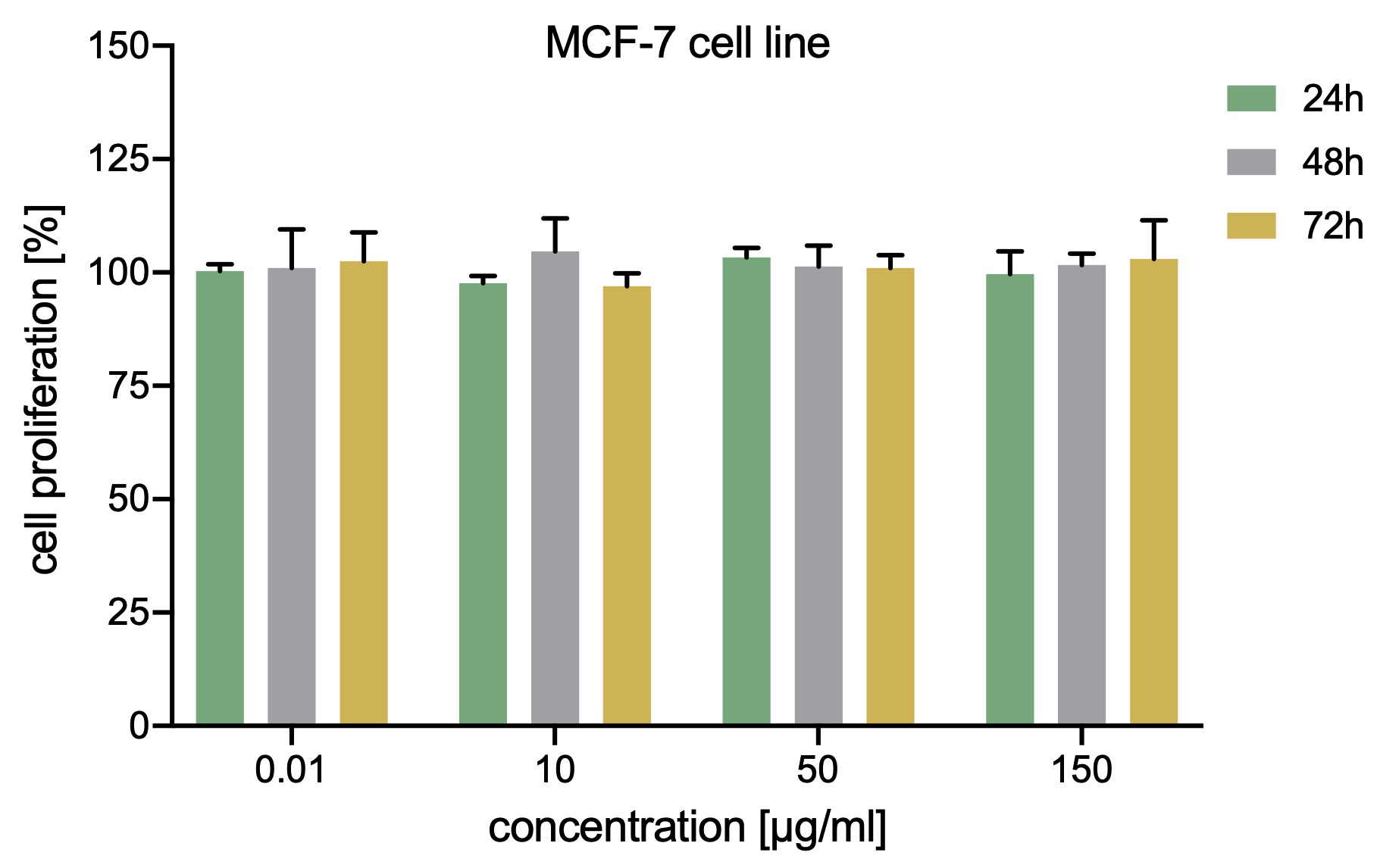

Supplement: Supplementary file 1 [file molecules-25-02884-s001.zip › supplementary materials/Figure S12.jpg]

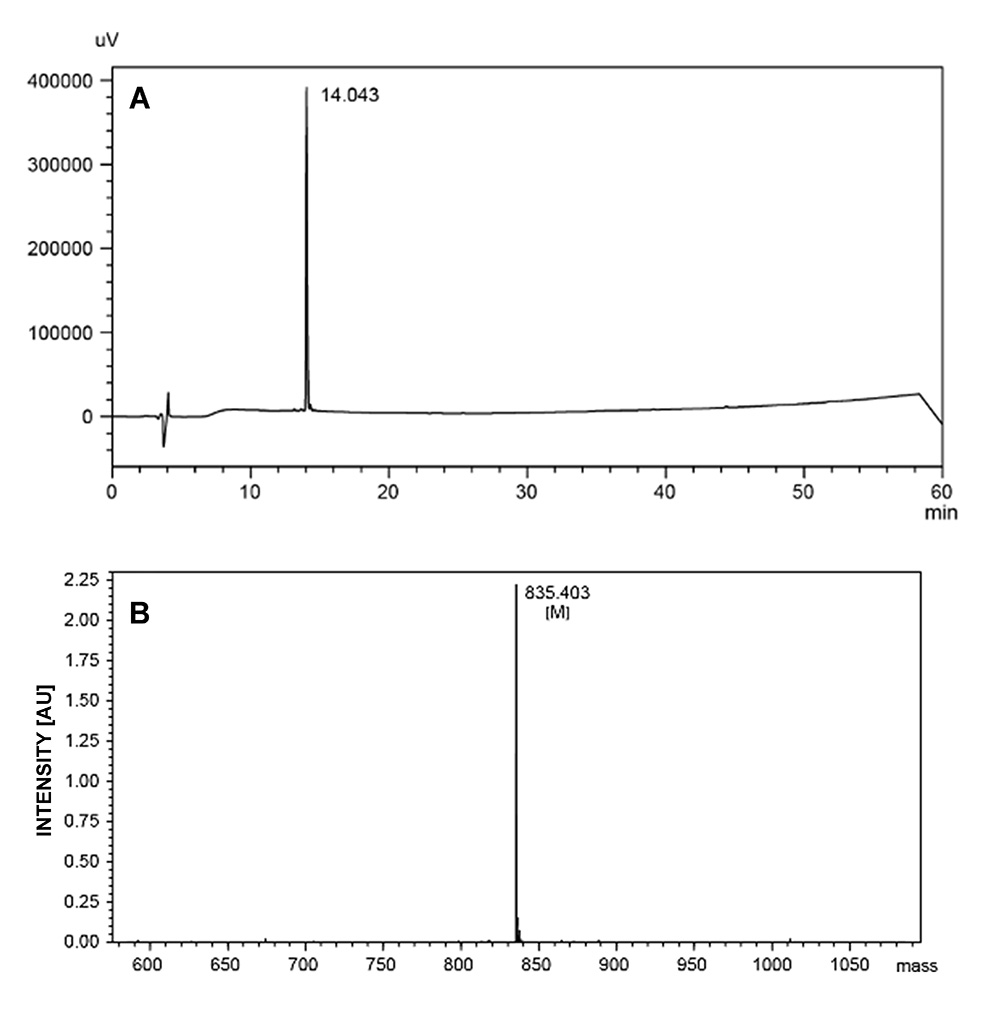

Supplement: Supplementary file 1 [file molecules-25-02884-s001.zip › supplementary materials/Figure S13.jpg]

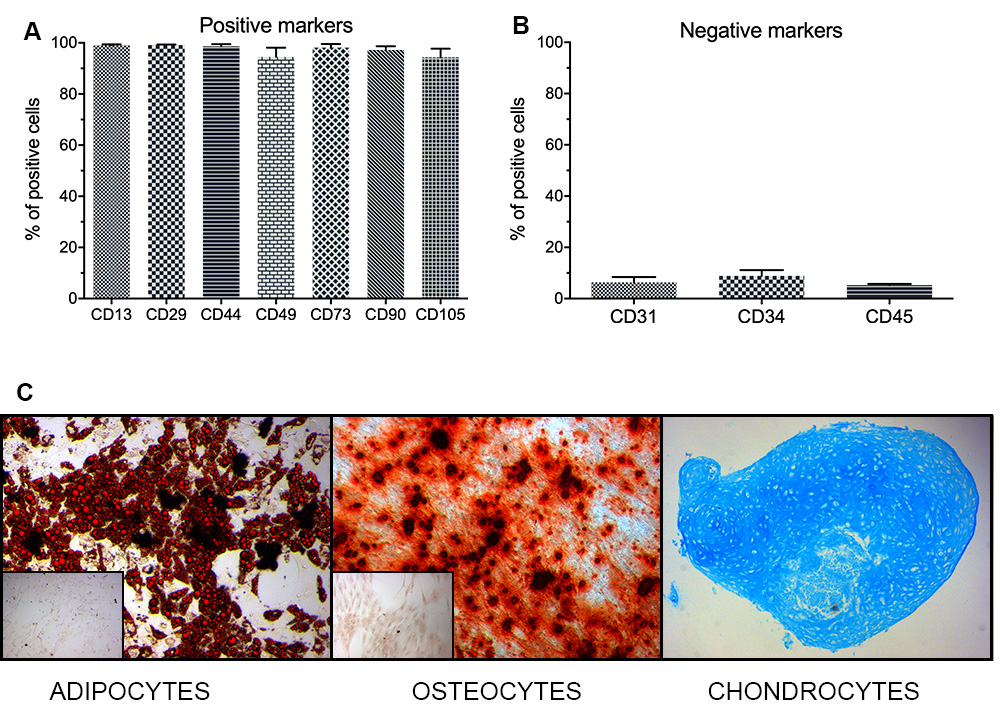

Supplement: Supplementary file 1 [file molecules-25-02884-s001.zip › supplementary materials/Figure S14.jpg]

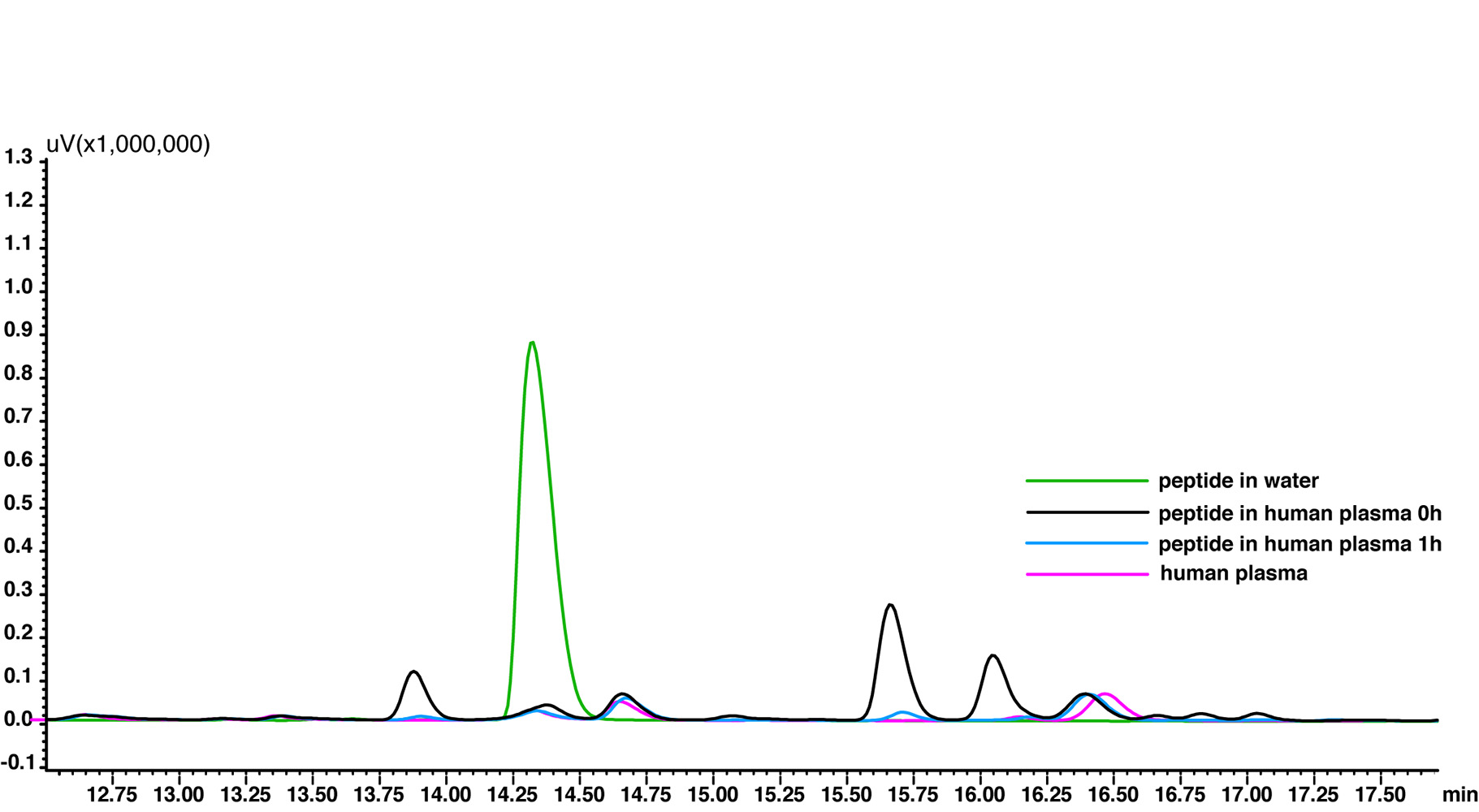

Supplement: Supplementary file 1 [file molecules-25-02884-s001.zip › supplementary materials/Figure S2.jpg]

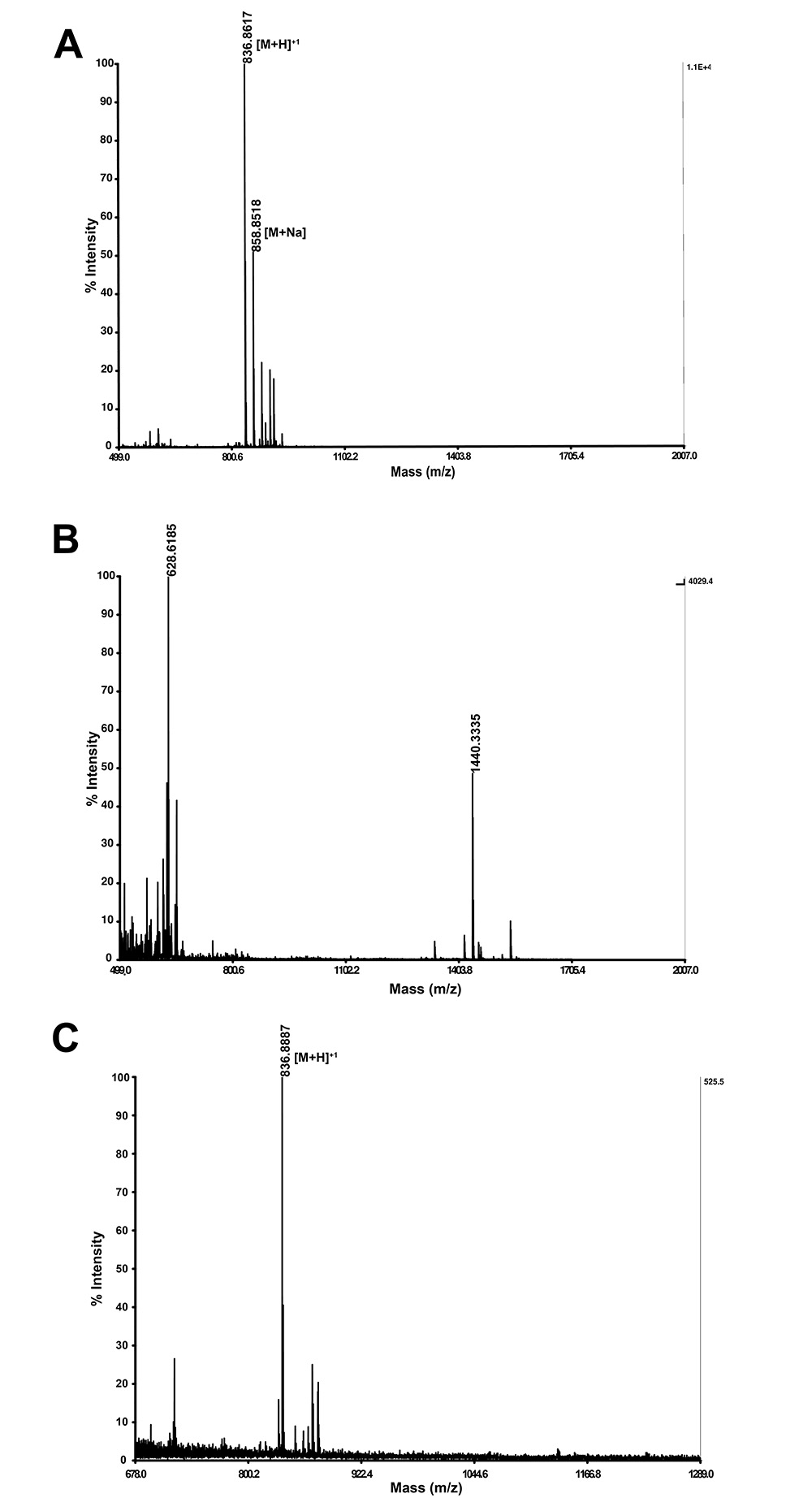

Supplement: Supplementary file 1 [file molecules-25-02884-s001.zip › supplementary materials/Figure S3.jpg]

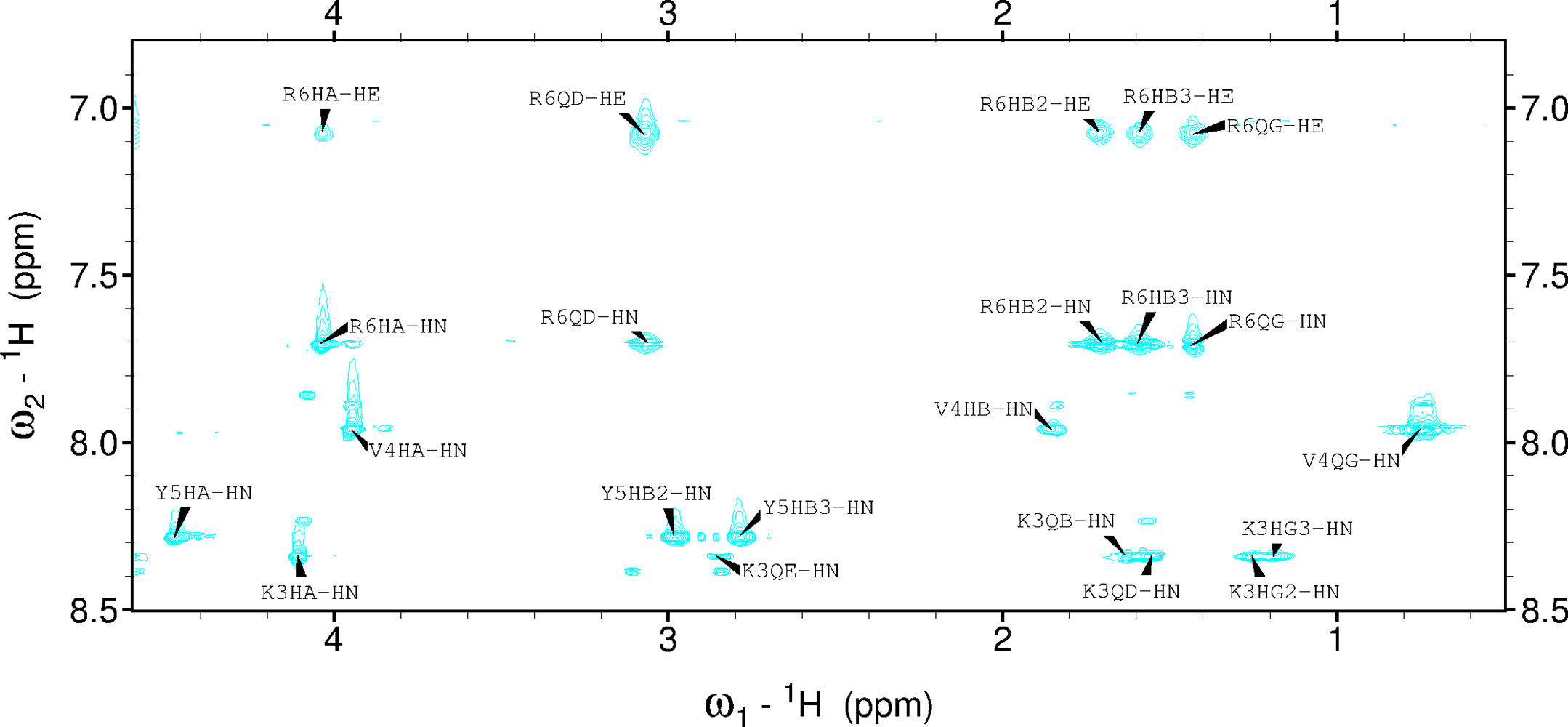

Supplement: Supplementary file 1 [file molecules-25-02884-s001.zip › supplementary materials/Figure S4.jpg]

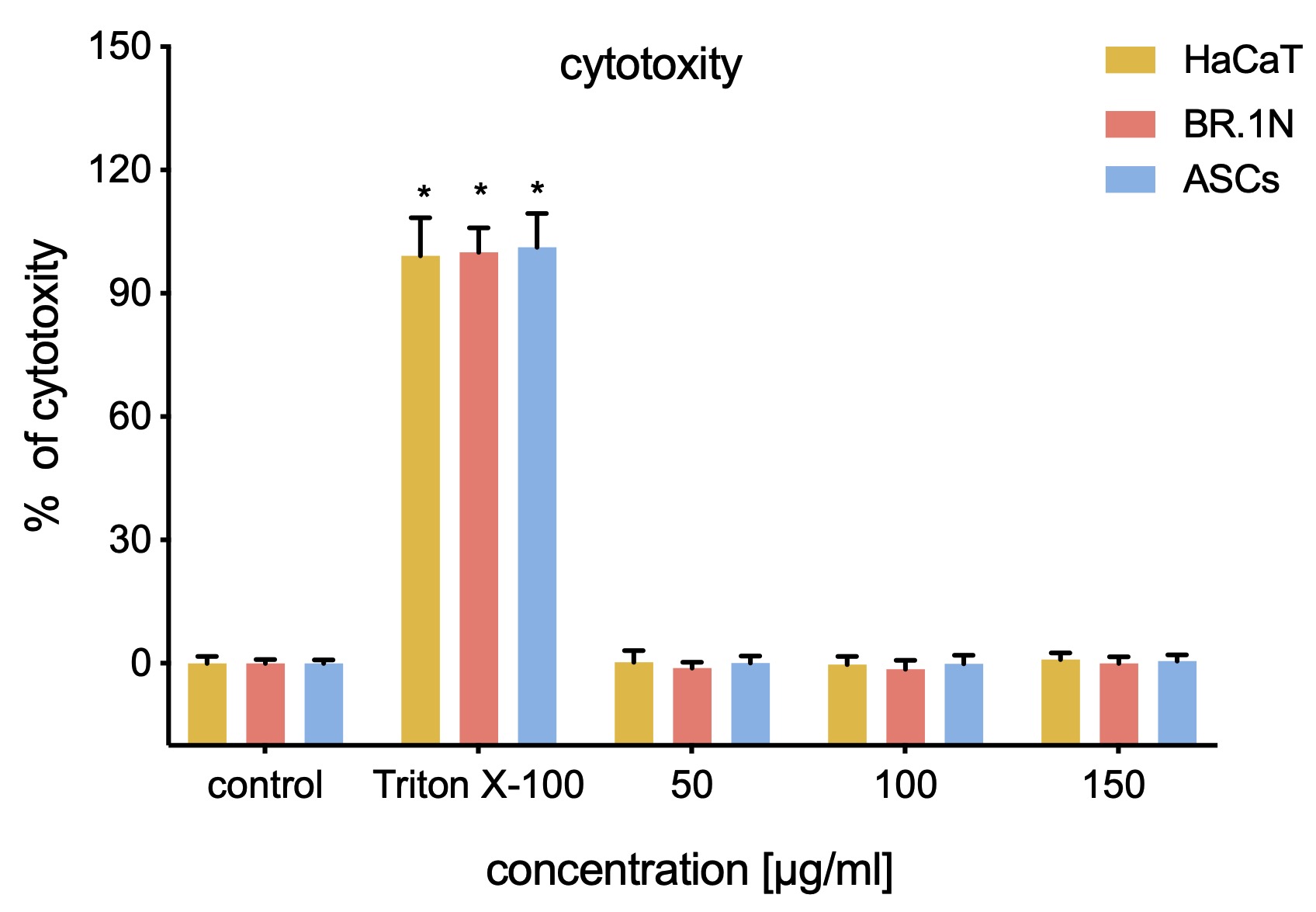

Supplement: Supplementary file 1 [file molecules-25-02884-s001.zip › supplementary materials/Figure S5.jpg]

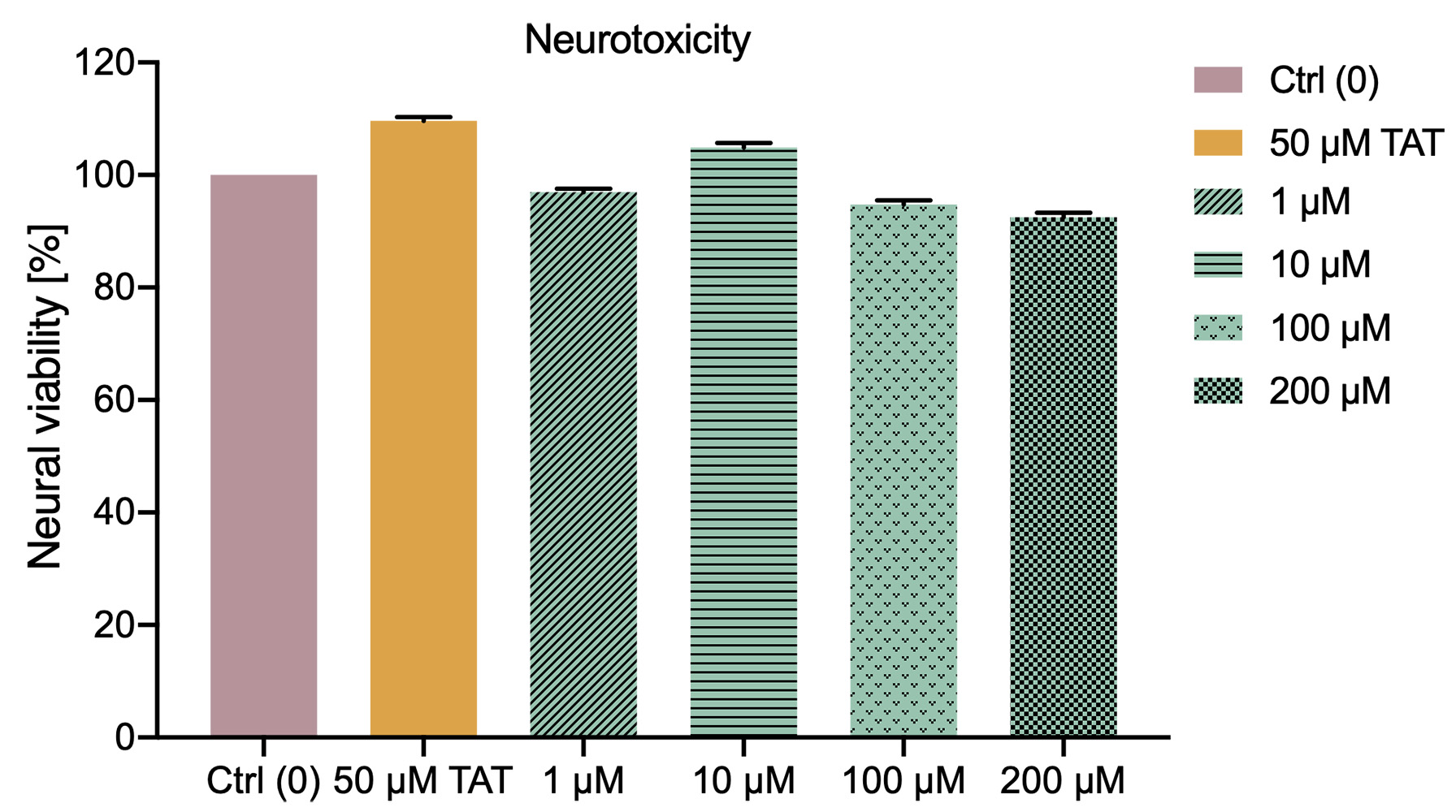

Supplement: Supplementary file 1 [file molecules-25-02884-s001.zip › supplementary materials/Figure S6.jpg]

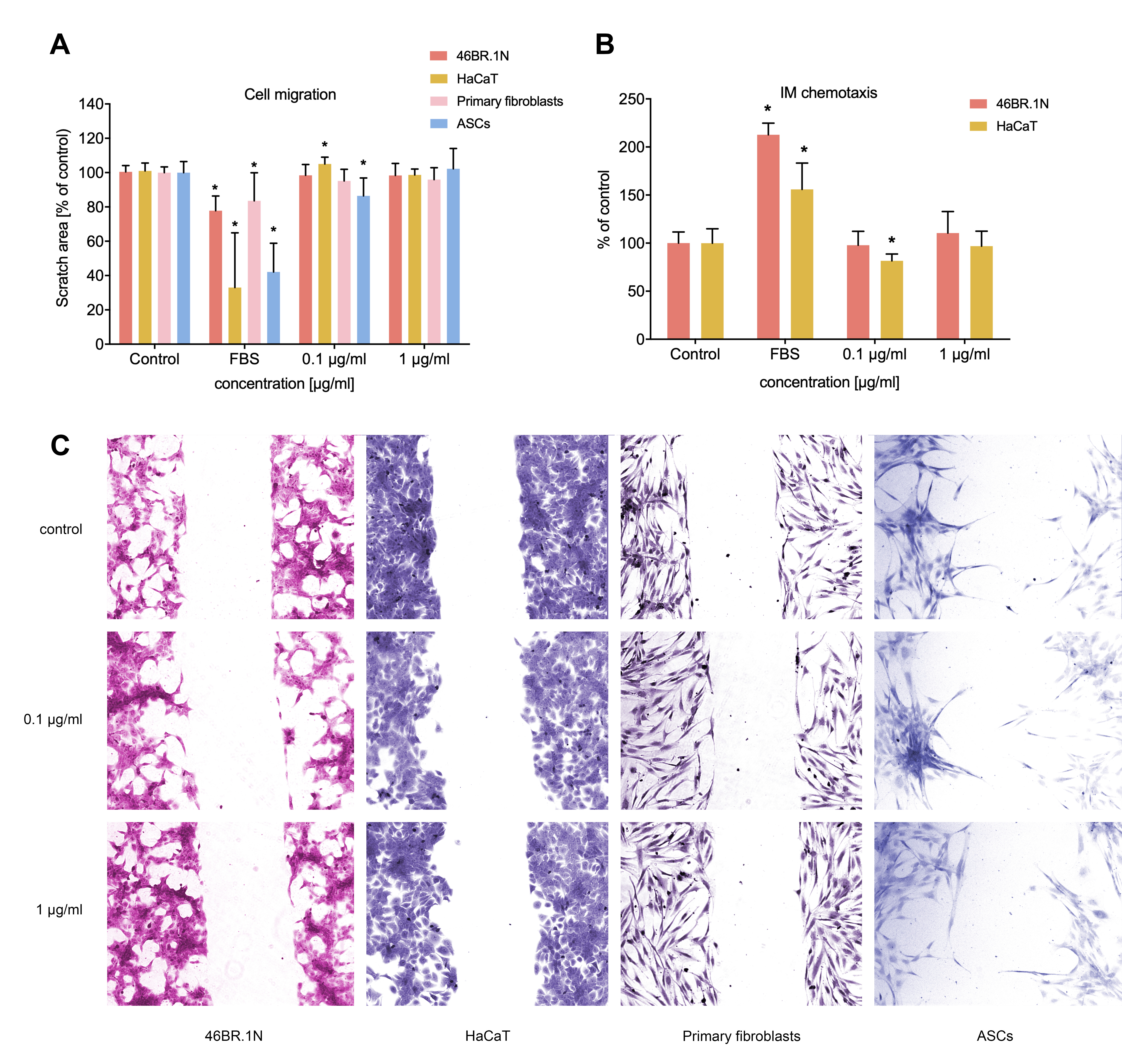

Supplement: Supplementary file 1 [file molecules-25-02884-s001.zip › supplementary materials/Figure S7.jpg]

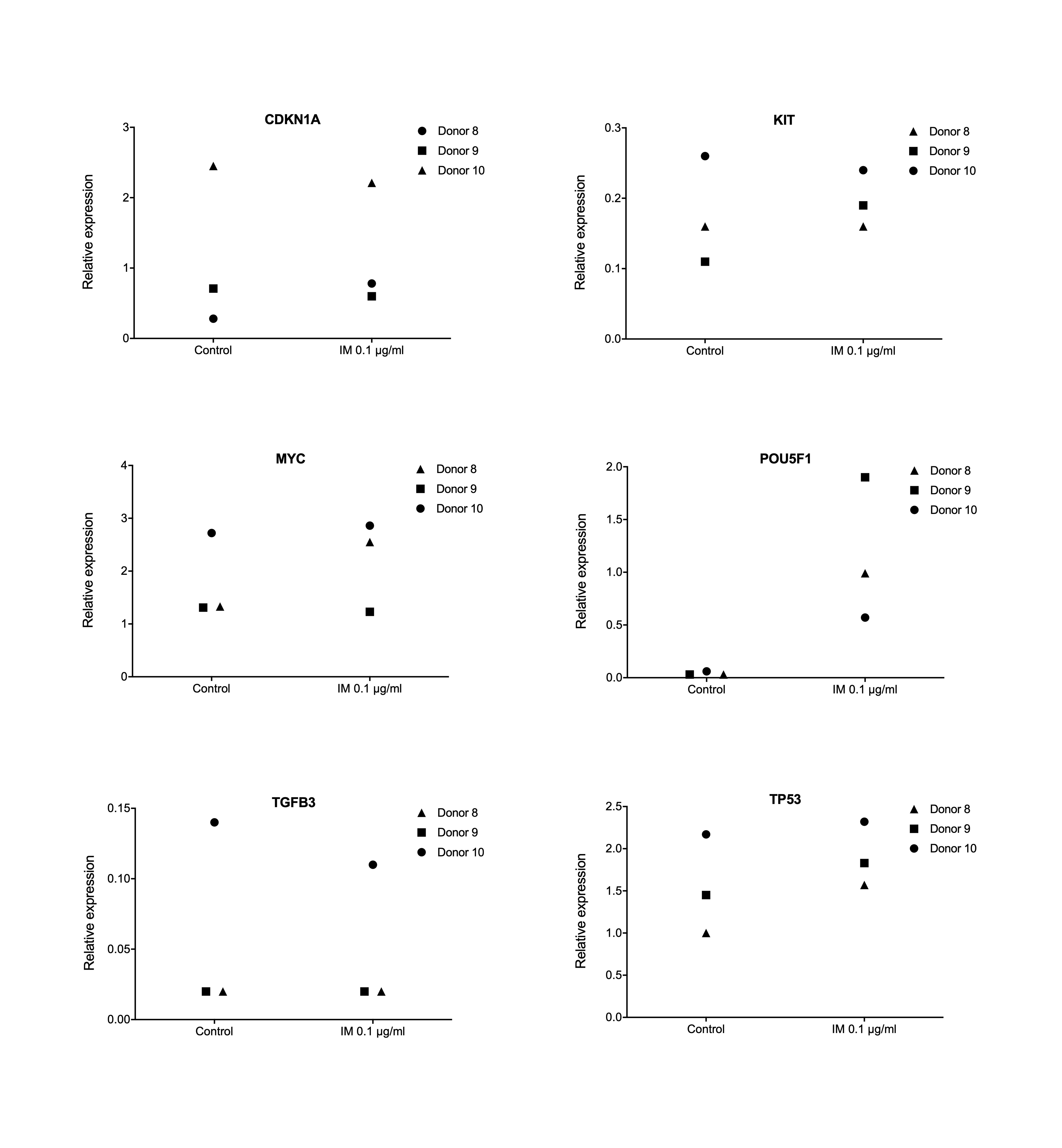

Supplement: Supplementary file 1 [file molecules-25-02884-s001.zip › supplementary materials/Figure S8a.jpg]

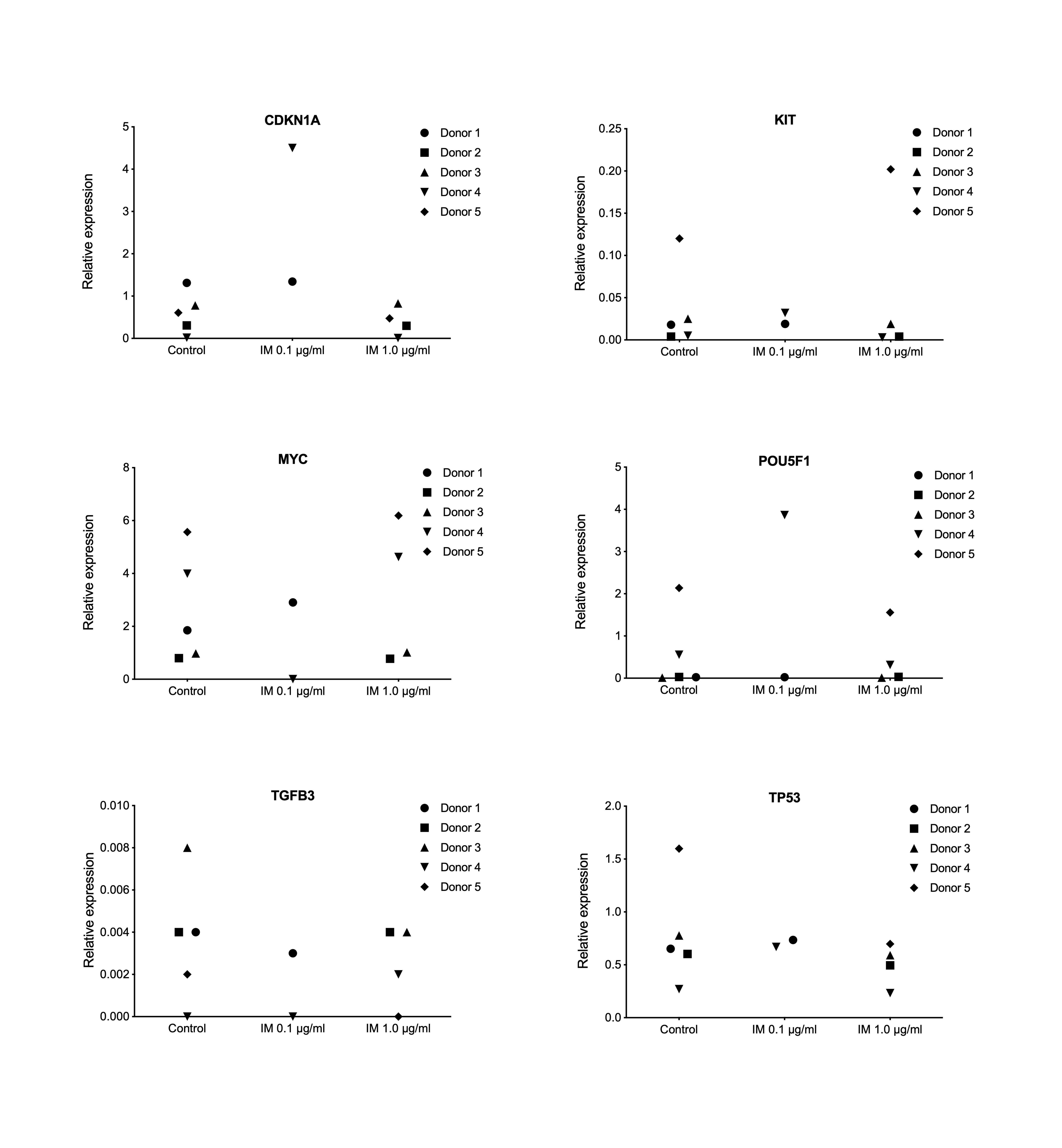

Supplement: Supplementary file 1 [file molecules-25-02884-s001.zip › supplementary materials/Figure S8b.jpg]

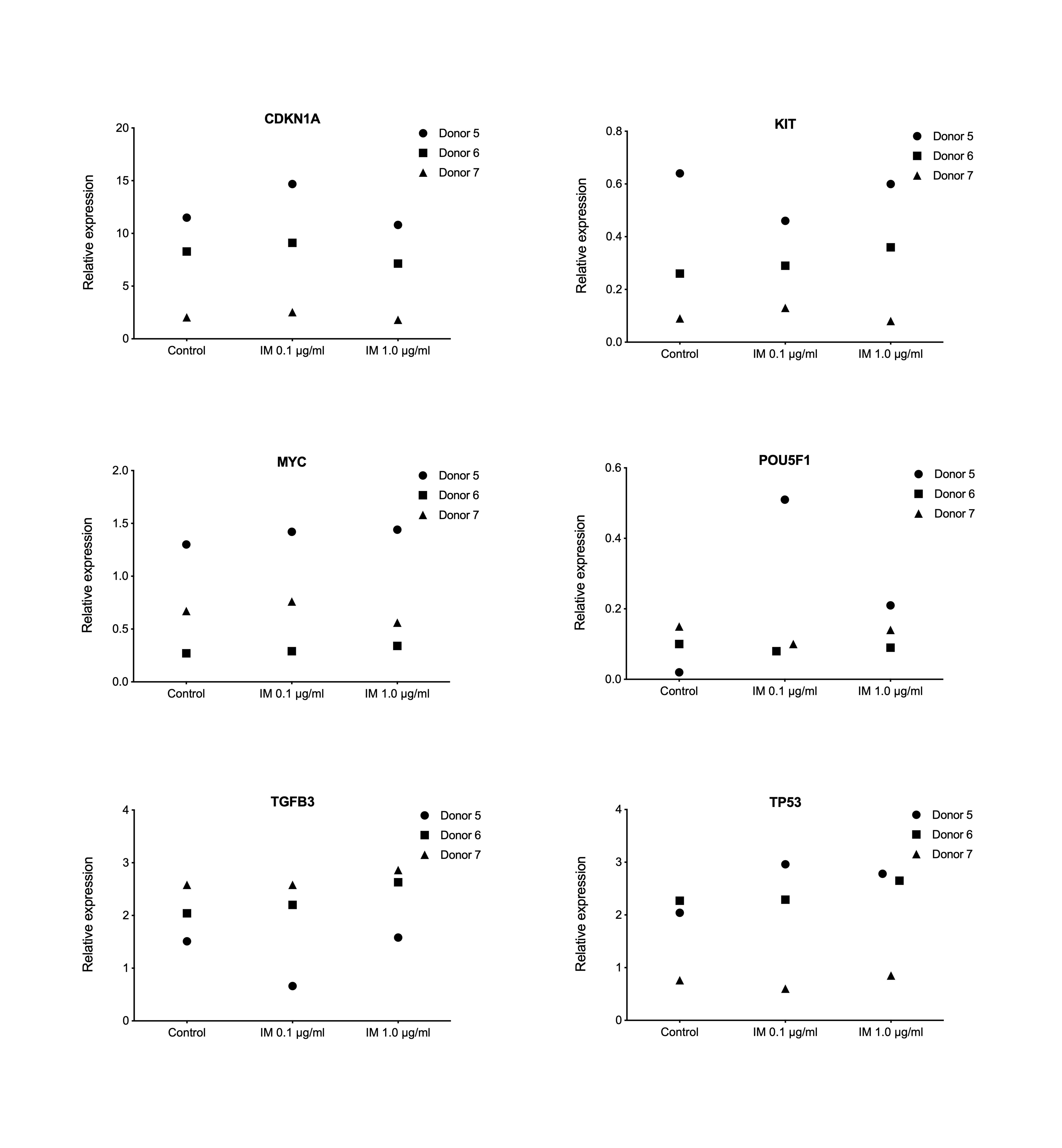

Supplement: Supplementary file 1 [file molecules-25-02884-s001.zip › supplementary materials/Figure S8c.jpg]

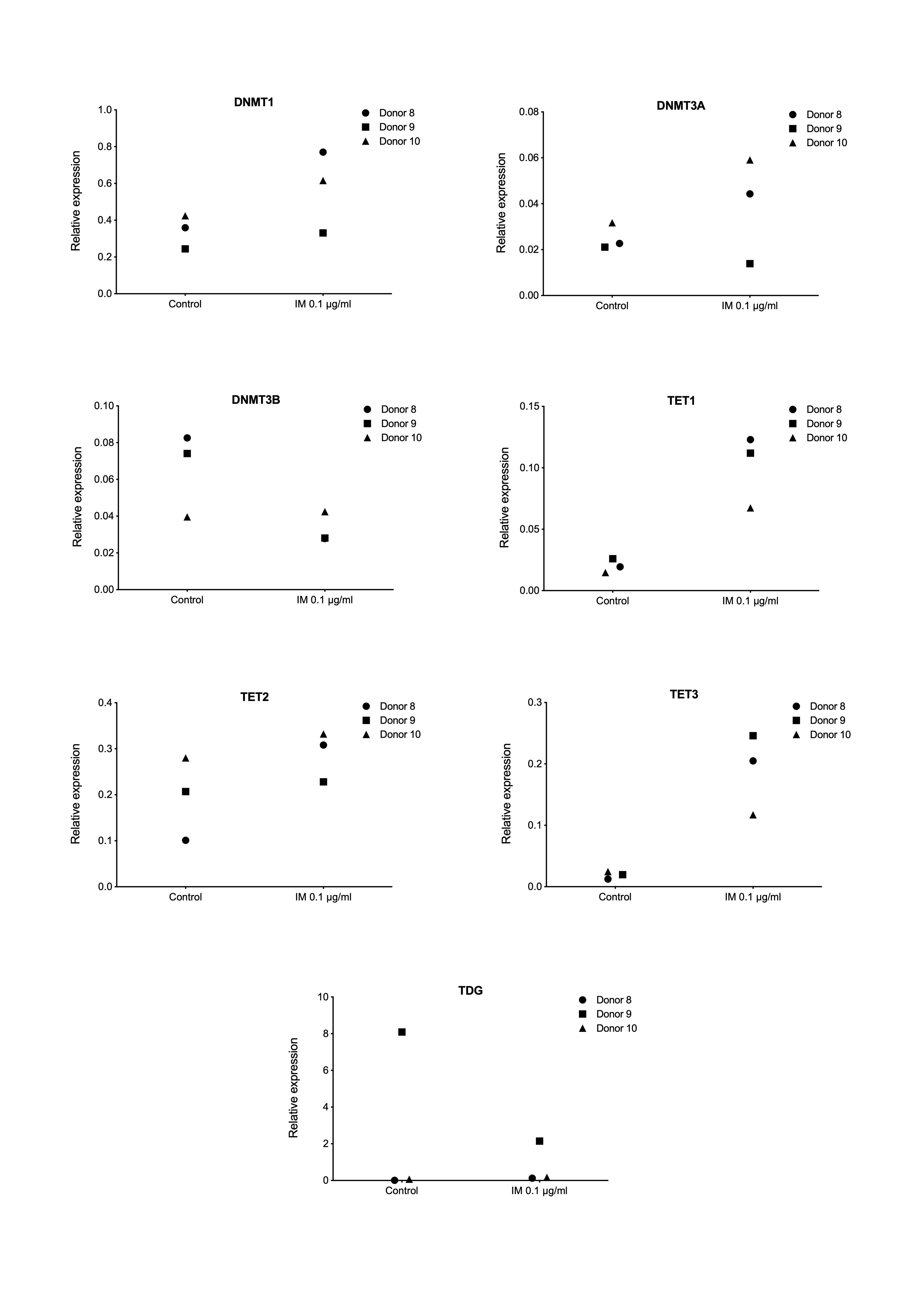

Supplement: Supplementary file 1 [file molecules-25-02884-s001.zip › supplementary materials/Figure S9.jpg]
